# Plasmodium falciparum GAP40 Plays an Essential Role in Merozoite Invasion and Gametocytogenesis

**DOI:** 10.1128/spectrum.01434-23

**Published:** 2023-05-30

**Authors:** Lu He, Yue Qiu, Geping Pang, Siqi Li, Jingjing Wang, Yonghui Feng, Lumeng Chen, Liying Zhu, Yinjie Liu, Liwang Cui, Yaming Cao, Xiaotong Zhu

**Affiliations:** a Department of Immunology, College of Basic Medical Sciences, China Medical University, Shenyang, Liaoning, China; b Department of Cardiovascular Ultrasound, The First Hospital of China Medical University, Shenyang, Liaoning, China; c Department of Laboratory Medicine, the First Hospital of China Medical University, Shenyang, Liaoning, China; d National Clinical Research Center for Laboratory Medicine, Shenyang, Liaoning, China; e College of Public Health, University of South Florida, Tampa, Florida, USA; National Institutes of Health

**Keywords:** GAP40, *Plasmodium falciparum*, glideosome, invasion, phosphorylation

## Abstract

Cyclic invasion of red blood cells (RBCs) by *Plasmodium* merozoites is associated with the symptoms and pathology of malaria. Merozoite invasion is powered actively and rapidly by a parasite actomyosin motor called the glideosome. The ability of the glideosome to generate force to support merozoite entry into the host RBCs is thought to rely on its stable anchoring within the inner membrane complex (IMC) through membrane-resident proteins, such as GAP50 and GAP40. Using a conditional knockdown (KD) approach, we determined that PfGAP40 was required for asexual blood-stage replication. PfGAP40 is not needed for merozoite egress from host RBCs or for the attachment of merozoites to new RBCs. PfGAP40 coprecipitates with PfGAP45 and PfGAP50. During merozoite invasion, PfGAP40 is associated strongly with stabilizing the expression levels of PfGAP45 and PfGAP50 in the schizont stage. Although PfGAP40 KD did not influence IMC integrity, it impaired the maturation of gametocytes. In addition, PfGAP40 is phosphorylated, and mutations that block phosphorylation of PfGAP40 at the C-terminal serine residues S370, S372, S376, S405, S409, S420, and S445 reduced merozoite invasion efficiency. Overall, our findings implicate PfGAP40 as an important regulator for the gliding activity of merozoites and suggest that phosphorylation is required for PfGAP40 function.

**IMPORTANCE** Red blood cell invasion is central to the pathogenesis of the malaria parasite, and the parasite proteins involved in this process are potential therapeutic targets. Gliding motility powers merozoite invasion and is driven by a unique molecular motor termed the glideosome. The glideosome is stably anchored to the parasite inner membrane complex (IMC) through membrane-resident proteins. In the present study, we demonstrate the importance of an IMC-resident glideosome component, PfGAP40, that plays a critical role in stabilizing the expression levels of glideosome components in the schizont stage. We determined that phosphorylation of PfGAP40 at C-terminal residues is required for efficient merozoite invasion.

## INTRODUCTION

Despite considerable control efforts, malaria remains a significant global public health burden, causing 627,000 deaths in 2020 (1). Of the five *Plasmodium* species that cause malaria in humans, Plasmodium falciparum accounts for the most malaria-attributed deaths. Current malaria prevention and cure practices rely heavily on antimalarial drugs, with artemisinin-based combination therapies being the first-line choice (2, 3). However, new therapies are urgently needed due to the emergence and spread of parasites resistant to artemisinin derivatives (4–6). The malaria parasite has a complex life cycle which involves multiple morphologically distinct developmental stages within the vertebrate host and mosquito vector. During parasite development, only the asexual blood cycle is associated with the disease symptoms and pathology (7). To complete the intraerythrocytic developmental cycle (IDC), merozoites invade red blood cells (RBCs) and develop through ring, trophozoite, and schizont stages to form 16 to 32 progeny merozoites within the mature schizont. The rupture of schizonts releases the merozoites, which invade new RBCs, and the resulting increasing burden of infection causes the symptoms of malaria (8). The recognition and mechanical invasion of RBCs by merozoites involves a repertoire of unique parasite proteins, which may be targets for new therapeutic approaches for malaria intervention.

*Plasmodium* is within the protozoan phylum Apicomplexa, of which members utilize a substrate-dependent mechanism, termed gliding motility, to invade target host cells (9). The gliding motility of *Plasmodium* merozoites and other apicomplexan motile stages is generated by an actomyosin motor, referred to as the glideosome (10). A core component of this motor is an unconventional class XIV myosin, myosin A (MyoA) (11, 12). Myosin tail interacting protein (MTIP) binds to the tail domain of MyoA and is proposed to serve as a lever arm for the motor (13, 14). Inhibitors, nanobodies, or peptides that directly target or interrupt the MyoA-MTIP interaction can block the merozoite invasion of RBCs (15–20). During early intracellular parasite development, the MyoA-MTIP complex initially interacts with the C terminus of the glideosome-associated protein 45 (GAP45) to form a soluble precomplex (MyoA-MTIP-GAP45) in the parasite cytosol (21, 22). The GAP45 C terminus then attaches the actomyosin motor complex to the inner membrane complex (IMC) through an interaction with an integral membrane protein of the IMC, GAP50 (23, 24). GAP45 is essential for correctly targeting the motor complex to the IMC, and its deletion impairs merozoite invasion (25). The N-terminal myristoylation and palmitoylation of GAP45 anchor the protein to the plasma membrane (PM) (22, 25, 26). GAP40 is another component of the glideosome (27), and in the apicomplexan Toxoplasma gondii, GAP50 and GAP40 are essential, such that depletion of either protein leads to morphological abnormality, impaired IMC integrity, and failure of the glideosome assembly (21, 28). GAP50 is essential in P. falciparum and in the rodent malaria model Plasmodium berghei (29, 30); while GAP40 has been shown to be essential in P. berghei (31, 32). These IMC-resident transmembrane proteins firmly anchor the glideosome to the IMC membrane, which is critical to the invasion and motility of the merozoite. When MyoA displaces the actin filaments rearward, the parasite moves forward to enter the host erythrocyte (33). Therefore, understanding the precise roles of IMC-resident transmembrane proteins, such as PfGAP40, in the invasion process may reveal new therapeutic targets.

Phosphoproteomic studies revealed that phosphorylation occurs on many glideosome components and plays a crucial role in the assembly and regulation of glideosome activity (34, 35). A major glideosome component, PfGAP45, was identified *in vitro* as a substrate for protein kinase B (PKB) and calcium-dependent protein kinase 1 (CDPK1) (26, 36, 37). *In vivo* assays showed that during schizogony, CDPK1 phosphorylated PfGAP45 at amino acids 81 to 96 and 141 to 155 and PfMTIP at serines 47 and 51 (37). CDPK1 has many targets that play critical regulator roles in invasion, and a study by Kumar et al. (38) showed that the CDPK1 kinase inhibitor K252a could ablate the phosphorylation of these sites on PfGAP45 and PfMTIP (37). This inhibition may cause an impairment of the merozoite invasion of RBCs, possibly by modulating the interaction between the two glideosome proteins. In merozoites, two additional sites within PfGAP45, namely, serines 149 and 156, are substrates of PfPKG (39). In T. gondii tachyzoites, phosphorylation of serine 163 and/or 167 of GAP45 regulates the assembly of the premotor complex to the IMC; possibly by preventing a premature insertion of the motor complex into the IMC during daughter cell formation (40). MyoA contains several phosphorylation sites (41), and there is evidence that the phosphorylation of serine 19 is under the control of PKG during merozoite invasion (39). Additionally, mutations of the major phosphorylation residues in T. gondii MyoA impact parasite egress from the host cell (42). High-throughput phosphoproteomic approaches identified several residues in PfGAP40 as phosphorylation targets of PfCRK4 during schizogony (43), and residues 448 and 449 on PbGAP40 are targets of CDPK4 (44). However, the extent to which phosphorylation of C-terminal residues is important for PfGAP40 function remains elusive.

Here, we used a CRISPR-Cas9-mediated conditional knockdown (KD) approach to determine that PfGAP40 is critical for RBC invasion and acts as a mediator for properly tethering PfGAP45 to the IMC. Moreover, our results show that PfGAP40 contributes to the regulation of gametocyte maturation. Using targeted mutagenesis, we showed that phosphorylation of PfGAP40 at C-terminal residues plays a role in merozoite invasion. This study demonstrates the significance of PfGAP40 in two developmental processes of the malaria parasite—the invasion of the RBC and gametocytogenesis.

## RESULTS

### PfGAP40 is a multiple transmembrane glideosome protein which is predominantly expressed in schizonts.

PfGAP40 (PF3D7_0515700) is a multitransmembrane (TM) domain glideosome protein and contains 19 phosphorylation sites identified by phosphoproteomic analyses (43, 45–48). Other features include a putative palmitoylation site (Cys142), three ubiquitin-binding sites (Lys 2, Lys 5, and Lys 346), and a low complexity region (359 to 378 amino acids [aa]) within the C terminus. PfGAP40 is conserved orthologously across the genus *Plasmodium* and in other apicomplexan parasites, such as *Toxoplasma*, *Cystoisospora*, *Besnoitia*, and *Babesia*, but is not found in nonapicomplexan genomes, suggesting that it performs vital apicomplexan-specific functions (see Fig. S1A and B in the supplemental material). We generated AlphaFold predictions in Uniprot, which yielded a putative three-dimensional (3D) structure of the PfGAP40 protein with transmembrane regions exhibiting good confidence scores (per-residue confidence score [pLDDT], >90) (Fig. S1C).

**FIG 1:**
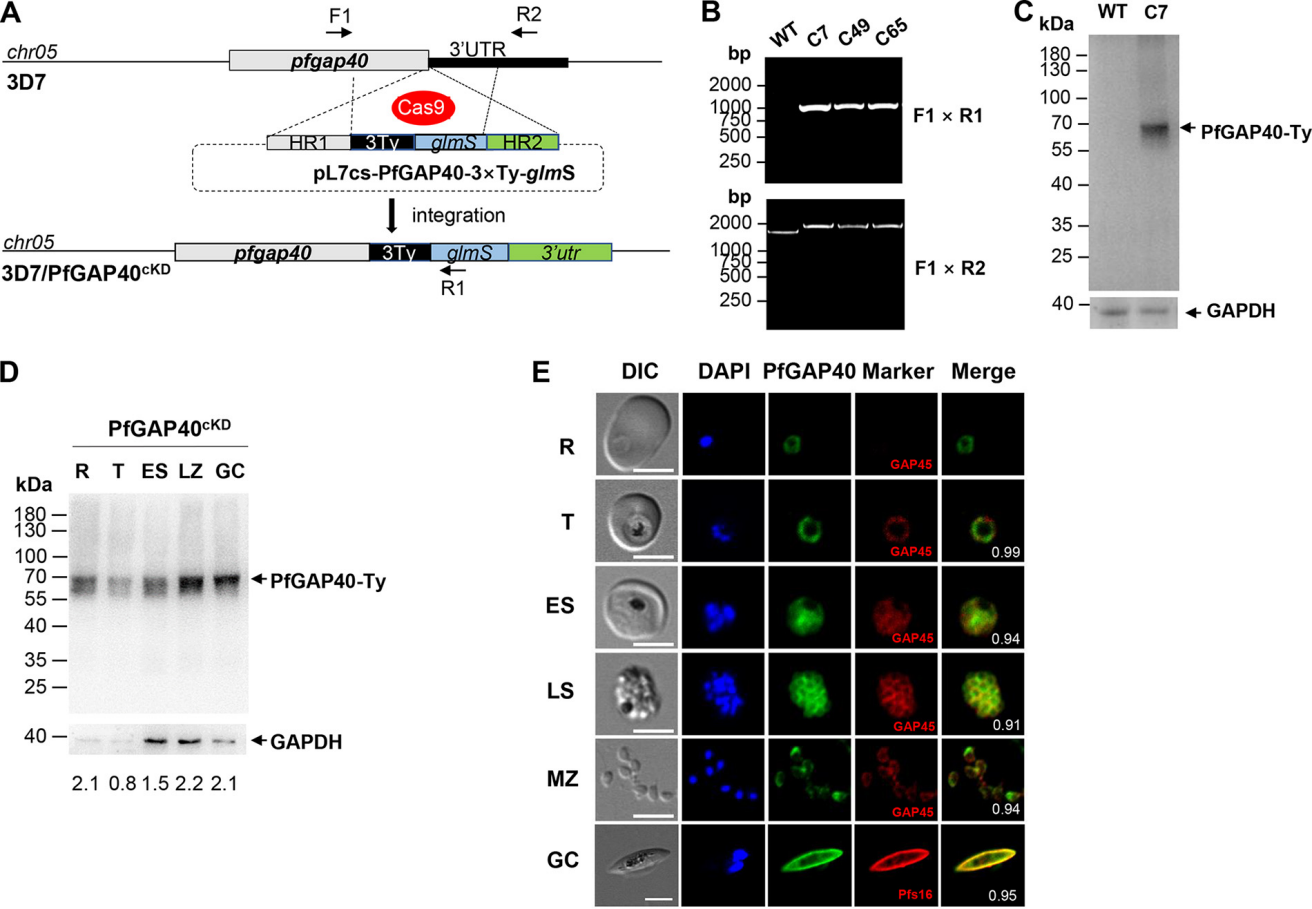
Expression and localization of PfGAP40 in Plasmodium falciparum. (A) Schematic representation of the Ty-*glmS* ribozyme tagging of GAP40 by the CRISPR-Cas9 system into the *gap40* locus. The ribozyme is inserted after the coding region within the 3′ UTR so that it is present in the expressed mRNA. Following the addition of the inducer, glucosamine (GlcN), which binds to the ribozyme, the mRNA self-cleaves resulting in degradation of the mRNA and knockdown of protein expression. The primers for diagnostic PCR are labeled. (B) Diagnostic PCR on genomic DNA showing the integration of PfGAP40-Ty-*glmS* (primers F1+R1: wild type [WT], null; PfGAP40^cKD^, 1,106 bp) and the unmodified gene locus (primers F1+R2: WT, 1,794 bp; PfGAP40^cKD^, 2,161 bp). (C) Western blot analysis of lysate from the PfGAP40^cKD^ mutant. Extracts of late-stage schizonts from 3D7 and the PfGAP40^cKD^ mutant C7 clone were immunoblotted with an anti-Ty antibody. GAPDH was used as a loading control. PfGAP40-Ty protein of ~68 kDa is indicated with a black arrow. (D) Western blot analysis showing a time course of PfGAP40-Ty expression in the intraerythrocytic development stages of P. falciparum. Extracts from tightly synchronized rings (R; 12 hpi), trophozoites (T; 24 hpi), early schizogony (ES; 36 hpi), late schizogony (LZ; 44 hpi), and gametocytes (GC) of the PfGAP40^cKD^ parasites were separated by SDS-PAGE under reducing conditions and subjected to immunoblotting with an anti-Ty monoclonal antibody (α-Ty) at a 1:500 dilution. Arrows indicate the recombinant PfGAP40-Ty protein. Anti-GAPDH antibody was used as a loading control. Representative results from three independent experiments are shown. (E) Costaining of PfGAP40-Ty with PfGAP45 in ring (R), trophozoite (T), early schizont (ES), late schizont (LZ), free merozoite (MZ), and gametocyte (GC) of P. falciparum. PfGAP40-Ty colocalized with PfGAP45 and Pfs16 around the periphery of segmented schizonts, free merozoites, and gametocytes. In the merged color image, the PfGAP40-Ty signal is green, the antibody specific for the PfGAP45 or Pfs16 protein is red, and the parasite nuclei are stained with 4′,6-diamino-2-phenylindole (DAPI) in blue. The differential inference contrast (DIC) images are also shown. The Pearson’s colocalization coefficient value of the Ty/GAP45 or Ty/Pfs16 in PfGAP40^cKD^ strain is shown in the right merged panel. Scale bars: 5 μm.

To characterize the expression profile and localization pattern of PfGAP40, we used a CRISPR-Cas9-based genome editing approach (49) to tag the C terminus of the endogenous *pfgap40* with a triple Ty epitope tag (3×Ty) and a *glmS* ribozyme (Fig. 1A). For all obtained clones, the expected integration of the 3×Ty-*glmS* sequence into the *pfgap40* locus was confirmed by genotyping PCR (Fig. 1B). One clone, PfGAP40^cKD^ clone 7 (C7), was selected for further analysis. Western blot analysis with the anti-Ty monoclonal antibody (MAb) confirmed the expression of the PfGAP40-Ty recombinant protein in the PfGAP40^cKD^ C7 clone, with a band of ~68 kDa detected under reducing conditions (Fig. 1C). We detected PfGAP40-Ty expression in PfGAP40^cKD^ parasite lysates from rings (12 h postinvasion [hpi]), trophozoites (24 hpi), early schizonts (36 hpi), late schizonts (40 hpi), and mixed gametocytes, although expression was relatively low in trophozoites (Fig. 1D). PfGAP40-Ty was highly expressed at the ring stage, suggesting that it may play a role following RBC invasion (Fig. 1D). Consistent with the Western blot results, immunofluorescence assays (IFAs) detected fluorescent signals of PfGAP40-Ty from rings to segregated schizonts, free merozoites, and gametocytes (Fig. 1E). In the ring to early schizont stages, the PfGAP40-Ty protein was localized in the cytosol, whereas it was localized to the periphery of merozoites in mature schizonts and free merozoites. PfGAP40 showed high-level colocalization with PfGAP45 (Pearson’s colocalization coefficient, >0.9), consistent with the presence of these proteins in a complex (Fig. 1E). Similarly, the PfGAP40 protein colocalized with Pfs16 in the periphery of stage IV gametocytes (Fig. 1E).

### PfGAP40 is essential for the IDC of P. falciparum.

**FIG 2:**
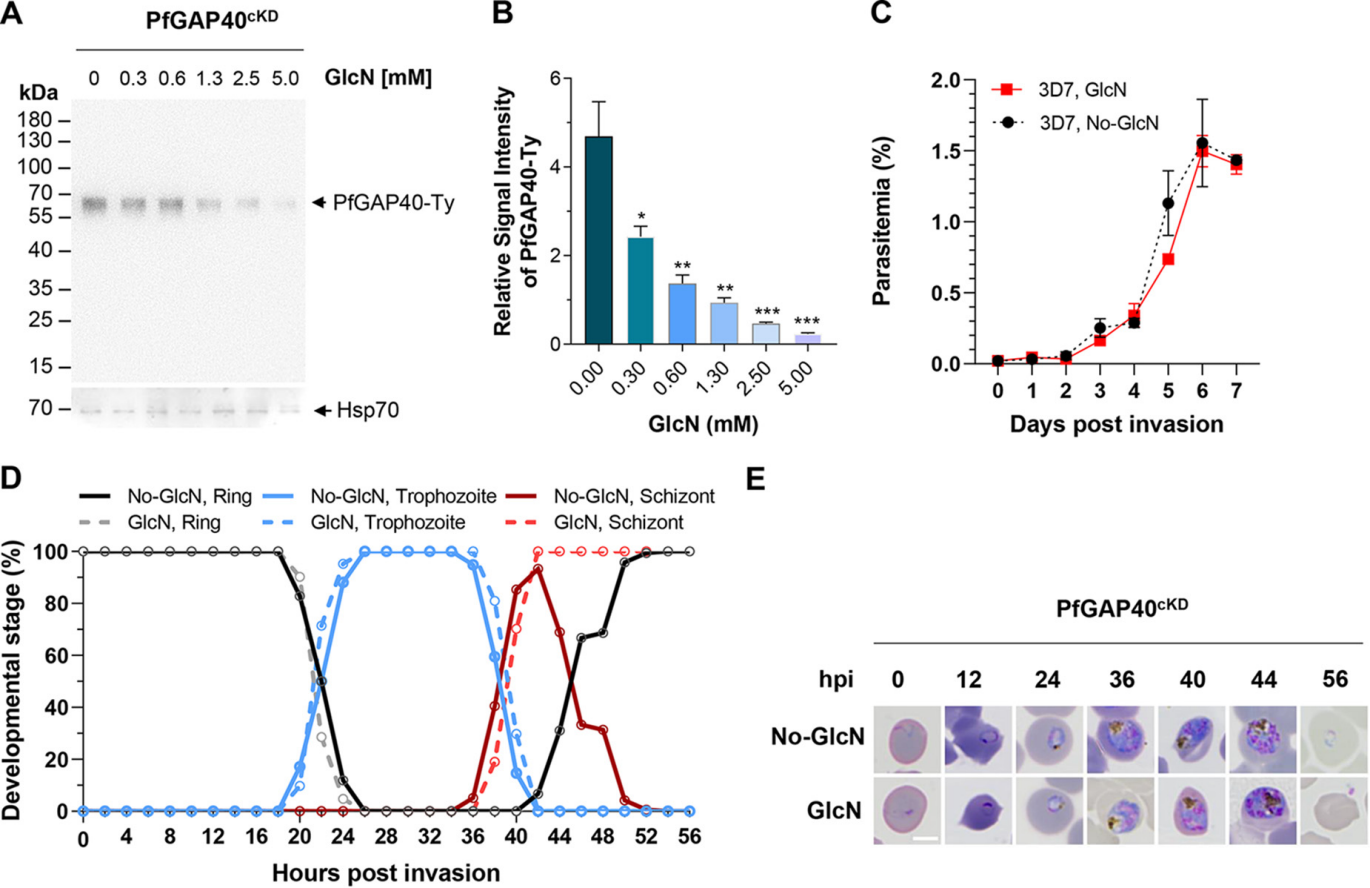
Conditional deletion of the PfGAP40-Ty protein and the effect on blood-stage growth. (A) Conditional deletion of PfGAP40-Ty protein in blood stages. Tightly synchronized P. falciparum GAP40^cKD^ cultures were treated with 0, 0.3, 0.6, 1.3, 2.5, and 5.0 mM GlcN at the ring stage (0 to 3 hpi) for 32 h. The schizont lysates from each GlcN-treated culture were analyzed for the expression level of PfGAP40-Ty using an anti-Ty MAb. Arrows indicate the position of the PfGAP40-Ty protein (molecular weight [MW], ~68 kDa). Protein loading per lane was verified using an anti-Hsp70 MAb. Representative results from three independent experiments are shown. (B) Band intensity analysis of PfGAP40-Ty. The signal intensity of PfGAP40-Ty protein was normalized to signals of Hsp70 protein using Image J software. All error bars indicate standard deviations (SD). Unpaired *t* test. *, *P < *0.05; **, *P < *0.01; ***, *P < *0.001. (C) Growth curves showing the proliferation of the 3D7 parasite following 2.5 mM GlcN treatment (GlcN) or mock treatment (No-GlcN). At each detection time point, the parasitemia was determined by Giemsa-stained blood thin smear, and growth was monitored over 7 days. Values are means and SD from 3 independent growth experiments. (D) Growth of No-GlcN and GlcN blood-stage PfGAP40^cKD^ parasites. Tightly synchronized blood-stage cultures of PfGAP40^cKD^ clone C7 were treated at the ring stage (3 hpi) with 2.5 mM GlcN (GlcN) or without GlcN (No-GlcN) for 56 h, and the parasitemia (mean ± SD) was detected every 2 h by Giemsa-stained blood thin smears. Results from 3 independent experiments are shown. (E) Various parasite stages were counted at the indicated times in both GlcN-treatment (GlcN) or mock-treatment (No-GlcN) PfGAP40^cKD^ parasites.

Since *pfgap40* appeared essential for asexual growth (31), we attempted to study its functions by conditional KD using the *glmS* ribozyme system (50). The addition of glucosamine (GlcN) into the blood-stage culture at the ring stage (3 hpi) reduced the PfGAP40-Ty protein level in a dose-dependent manner, with a roughly 95% reduction of the fusion protein observed after incubation with 2.5 mM GlcN for ~36 hpi (Fig. 2A and B). We found that a 2.5 mM GlcN treatment did not influence the blood-stage replication of the parental 3D7 strain (Fig. 2C). These results demonstrate the successful KD of PfGAP40 with the ribozyme system.

To study the role of PfGAP40 in blood-stage development, we induced KD of PfGAP40 in tightly synchronized PfGAP40^cKD^ C7 parasites (2-h window) by 2.5 mM GlcN treatment at the early ring stage (3 hpi) and monitored the parasite growth for one invasion cycle (0 to 56 hpi). We found that GlcN-treated PfGAP40^cKD^ C7 parasites developed at almost the same rate as control parasites with no morphological abnormalities. All PfGAP40^cKD^ parasites developed to segregated schizonts at roughly 44 hpi, indicating that PfGAP40 is not essential for asexual growth through schizont maturation (Fig. 2D). However, no reinvaded rings were observed in the PfGAP40-Ty KD parasites in the following cycle (44 to 56 hpi), suggesting the presence of severe defects in merozoite egress or invasion (Fig. 2D and E).

### PfGAP40 is dispensable for egress but is required for RBC invasion.

**FIG 3:**
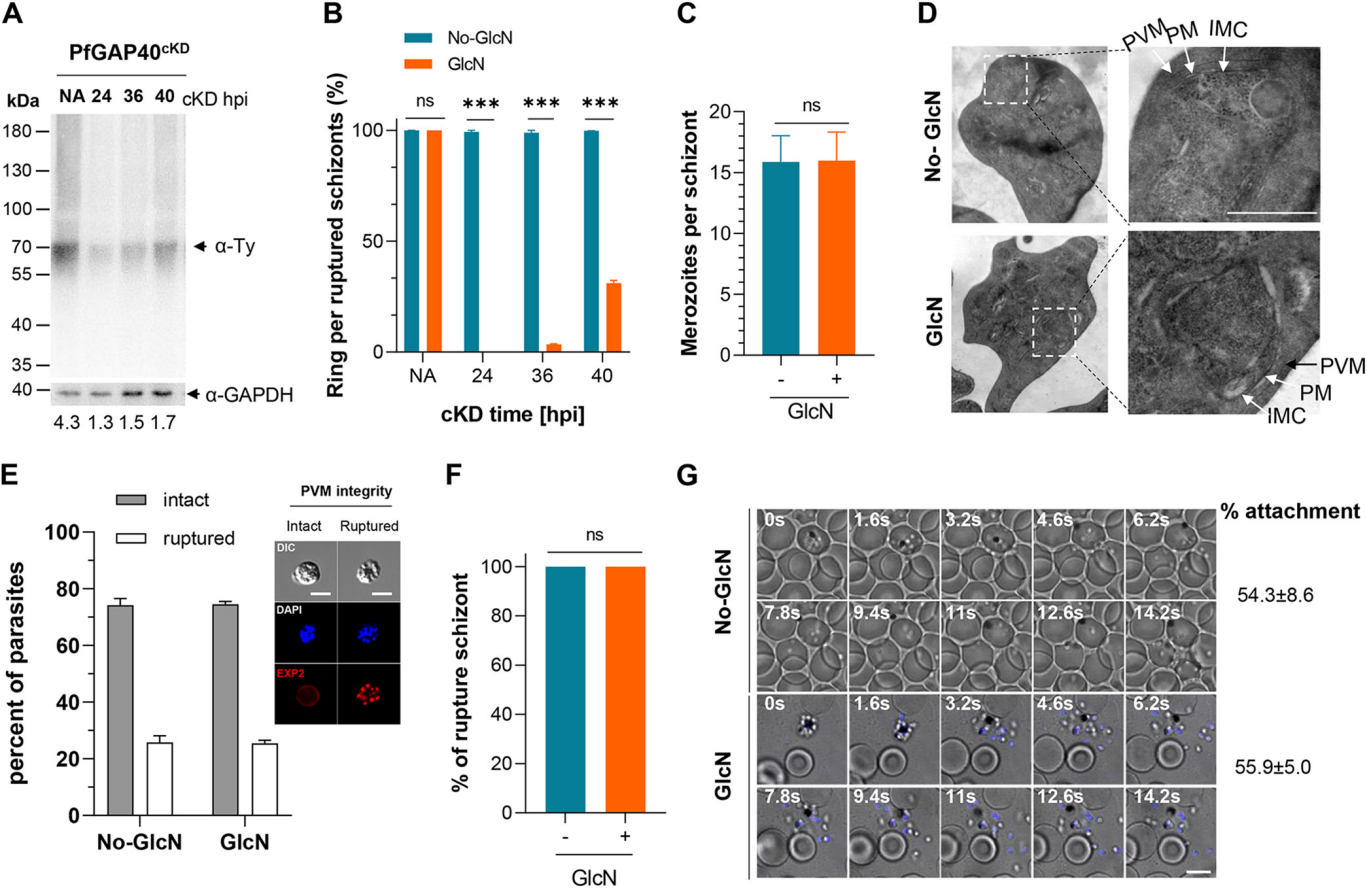
PfGAP40 function in egress to invasion of asexual-stage development. (A) Western blot analysis of parasite extracts from PfGAP40^cKD^ parasites with 2.5 mM GlcN treatment since 24, 36, 40, and 44 hpi. The GlcN-treated samples (GlcN-treated time points of 24 h, 36 h, 40 h, and 44 h) were collected at 44 hpi and assessed by immunoblot using an anti-Ty MAb (α-Ty). Representative of 2 experiments. Molecular mass is shown in kDa. NA, PfGAP40^cKD^ cultures treated without GlcN. GAPDH was used as a loading control. (B) Rings per ruptured schizonts. Values are means ± SD (*n* = 3 biological replicates). Unpaired *t* test. ns, not significant; ***, *P < *0.001. (C) Number of merozoites per matured schizont in No-GlcN and GlcN blood-stage PfGAP40^cKD^ parasites. ns, not significant. Values are means and SD from 3 independent experiments. (D) Electron microscopy of terminally developed PfGAP40^cKD^ parasites treated with or without GlcN at 3 hpi. In both images, the different membranes are indicated by arrows, as follows: parasitophorous vacuolar membrane (PVM), plasma membrane (PM), and inner membrane complex (IMC). Representative of 2 experiments. Scale bar: 1 μm. (E) PVM rupture at 45 hpi in tightly synchronized PfGAP40^cKD^ parasites treated with or without GlcN at 3 hpi. Right: immunofluorescence images of parasites with intact or ruptured PVMs. Left: proportion of infected cells exhibiting PVM rupture. PfGAP40^cKD^ parasites with or without GlcN treatment at 3 hpi were treated with E64 (50 μM) at 41 hpi, and the completion of cytokinesis was assessed with the parasite plasma membrane marker EXP2. Scale bars: 5 μm. Representative results are from 3 independent experiments are shown. Error bars indicate SD. Two-tailed *t* test was used for statistical analysis. (F) The percentages of ruptured schizonts in GlcN- or mock-treated PfGAP40^cKD^ parasites. Unpaired *t* test. ns, not significant. (G) Live imaging was performed on PfGAP40^cKD^ parasites treated with (GlcN) or without GlcN (No-GlcN). These selected snapshots from Movie S1 show a mock-treated PfGAP40^cKD^ merozoite successfully egressing, attaching to the RBC, deforming its membrane, and invading. PfGAP40^cKD^ parasites treated with GlcN attach to the RBC membrane (Movie S2) without invasion. Percentages of merozoites attached to RBCs in No-GlcN and GlcN groups are shown in the right panel.

To determine how long the incubation is needed to show an effect, we induced PfGAP40-Ty KD in trophozoites (24 hpi), early schizonts (36 hpi), and late schizonts (40 hpi) using 2.5 mM GlcN and measured the rings formed per ruptured schizont in the next cycle. We found that the initiation of the GlcN treatment at 24, 36, and 40 hpi reduced the PfGAP40-Ty level at 44 hpi by 70%, 65%, and 60%, respectively (Fig. 3A). Treatment at these three time points corresponded to a 100%, 96%, and 67% reduction in RBC reinvasion in the next cycle (Fig. 3B).

We compared schizogony between the PfGAP40-Ty KD and control parasites following GlcN treatment starting at 3 hpi. A comparable number of merozoites per schizont was observed in the GlcN-treated (15.88 ± 2.45) and control parasites (15.58 ± 2.02), indicating that PfGAP40 KD did not perturb the schizogony process (Fig. 3C). Transmission electron microscopy (TEM) showed that the PfGAP40 KD parasites displayed fine structures typical of normal schizont maturation, including intact RBC membranes, parasitophorous vacuole membranes (PVMs) that surround parasites, and individual parasite cells physically distinguished by the plasma membranes (PM), indicating that PfGAP40 KD did not interfere with the completion of cytokinesis (Fig. 3D).

To initiate RBC egress at the end of schizogony, the PVM and RBC membranes are sequentially ruptured following the activation of a PfSUB1-mediated proteolytic cascade (51). In segregated schizonts, intact or ruptured PVM can be visualized using an anti-EXP2 antibody which presents as a circular or fragmented pattern around the parasites. To examine whether PfGAP40 is required for PVM rupture, we performed IFA using an anti-EXP2 antibody in PfGAP40^cKD^ parasites. The proportions of the PfGAP40^cKD^ parasites with ruptured PVM were not significantly different between GlcN-treated (25.5%) and control groups (25.8%), suggesting that PfGAP40 is not required for PVM rupture (*P *= 0.86) (Fig. 3E). Furthermore, we observed that 100% of schizonts ruptured from RBC membranes in GlcN-treated PfGAP40^cKD^ parasites, indicating that PfGAP40 is dispensable for the egress event (Fig. 3F). Since the PfGAP40^cKD^ KD parasites could egress efficiently, we reasoned that a defect in merozoite invasion of the host RBC might most likely cause the proliferation failure. Using time-lapse microscopy, we found that compared with the control parasites (Fig. 3G; see Movie S1 in the supplemental material), GlcN treatment did not affect merozoite attachment to the RBC but completely blocked host cell entry (Fig. 3G; see Movie S2 in the supplemental material). To further separate the two functions, we used cytochalasin D (CytD), a small molecular inhibitor of actin polymerization that could block host cell entry but did not affect the RBC attachment of merozoites (52). The PfGAP40^cKD^ parasites with GlcN treatment exhibited a comparable percentage of RBC attachment (54.3% ± 8.6%) with mock-treated (55.9% ± 5.0%) PfGAP40^cKD^ parasites, confirming that PfGAP40 does not regulate the RBC attachment of merozoites (Fig. 3G). These results conclusively showed that PfGAP40 depletion impaired only merozoite entry into host RBCs.

### PfGAP40 plays a role in gametocyte maturation.

**FIG 4:**
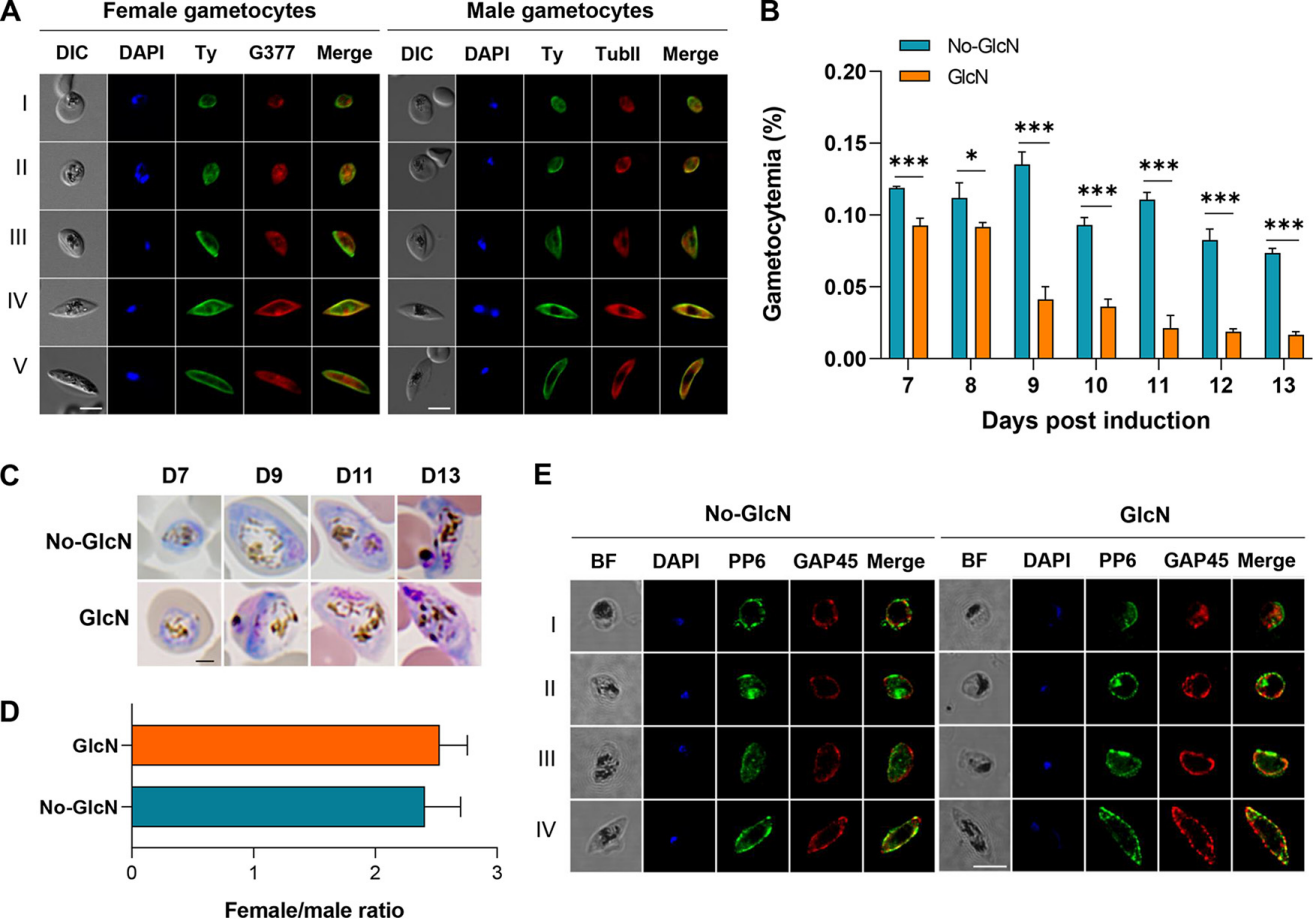
Conditional PfGAP40 knockdown in PfGAP40^cKD^ parasites impacts gametocytogenesis. (A) Localization of the PfGAP40 protein in female and male gametocytes. (B) Gametocytemia. Synchronous parasites were split (GlcN/No-GlcN) as sexual/asexual ring-stage parasites 24 h after the induction of sexual commitment in the preceding IDC. To eliminate asexual parasites, gametocytes were cultured in complete medium (CM) supplemented with 50 mM GlcNAc from day 1 to 6 of gametocytogenesis. Representative results from three biological replicates are shown. Error bars indicate SD. Unpaired *t* test. *, *P < *0.05; ***, *P < *0.001. (C) Representative images captured from Giemsa-stained thin blood smears showing the distinct morphology of stage I to V gametocytes cultured under PfGAP40-Ty-depleting (GlcN) and control conditions (No-GlcN) over 13 days of maturation. Scale bar: 5 μm. (D) Female:male gametocyte ratio in GlcN- or mock-treated PfGAP40^cKD^ parasites at day 11 postinduction. Representative results from three biological replicates are shown. Error bars indicate SD. (E) Representative IFA images of GlcN- or mock-treated PfGAP40^cKD^ gametocytes labeled with anti-PP6 (a marker for parasite plasma membrane), anti-GAP45 (a marker for the IMC), and DAPI. BF, bright field. Scale bar: 5 μm.

We observed the expression of the PfGAP40 protein in both male and female gametocytes (Fig. 4A). A previous study showed that glucosamine at 2.5 mM does not affect gametocyte development in wild-type parasites (53). Therefore, we next tested if the induction of PfGAP40 KD using 2.5 mM glucosamine affected gametocyte development in P. falciparum. Tightly synchronized schizont-stage cultures were started at 0.3% parasitemia and 6% hematocrit in 15 mL complete medium (54). After parasite reinvasion, the culture was split and cultured with or without GlcN treatment. For the first 6 days of the gametocyte culture, the culture medium was supplemented with 50 mM *N*-acetyl-d-glucosamine (GlcNac) to eliminate asexual parasites (55). Subsequently, we found that the gametocytemia of the PfGAP40 KD culture was significantly reduced compared with that of the control (Fig. 4B). Meanwhile, in Giemsa-stained blood thin smears, we detected apparent morphological abnormalities of PfGAP40 KD gametocytes in developmental stages III to V, with PfGAP40 KD gametocytes arrested at stage IV (Fig. 4C). However, the female-to-male ratio was not affected by PfGAP40 KD (Fig. 4D). Despite the morphological changes, both the IMC marker GAP45 and the PM protein PfPP6 remained localized to the periphery of the parasite, suggesting an integrity of IMC and PM in the PfGAP40 KD gametocytes (Fig. 4E). To confirm the role of PfGAP40 in gametocyte development, we generated a PfGAP40^cKD^ line (named NF54::PfGAP40^cKD^) using the NF54 strain, in which a quantitative and robust induction of gametocytogenesis is possible (see Fig. S2A in the supplemental material). The NF54::PfGAP40^cKD^ line was confirmed by PCR and Western blot (Fig. S2B and C). As expected, the asexual stage phenotype of the NF54::PfGAP40^cKD^ line upon PfGAP40 KD was comparable as detected in the 3D7::PfGAP40^cKD^ line (data not shown). Although the NF54::PfGAP40^cKD^ line treated with 2.5 mM GlcN showed no significant difference in the female-to-male ratio compared with the mock-treated line, there was a 25.5% to 54.4% reduction in gametocytemia in the NF54::PfGAP40^cKD^ parasites treated with 2.5 mM GlcN since day 7 post-gametocyte induction (Fig. S2D to E). Simultaneously, we found that PfGAP40 KD had a substantial impact on gametocyte maturation (Fig. S2F). Taken together, these results showed that PfGAP40 plays an essential role in gametocytogenesis.

### PfGAP40 KD reduces the expression level of PfGAP45 and PfGAP50 in the schizont stage.

**FIG 5:**
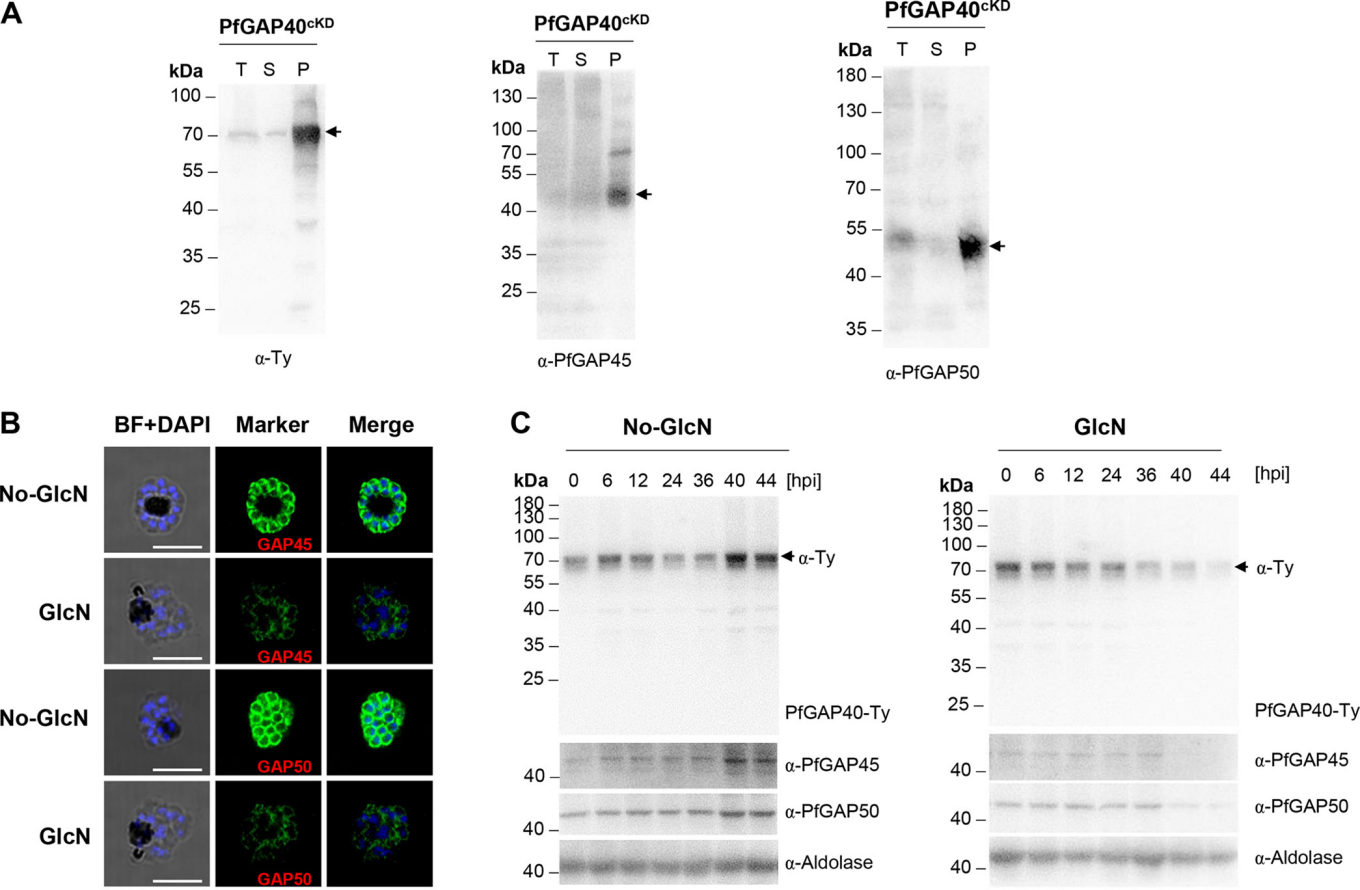
Loss of PfGAP40 affects targeting of PfGAP45 and PfGAP50 to the cytoskeletal IMC. (A) IP analysis of PfGAP40^cKD^ parasites. The PfGAP40^cKD^ parasites expressing PfGAP40-Ty were subjected to IP with the anti-Ty antibody, and the presence of PfGAP40-Ty (left), PfGAP45 (middle), and PfGAP50 (right) in the elution was checked by Western blotting. T, total protein; S, supernatant of co-IP analysis; P, pellet of co-IP analysis. Arrows indicate the relative endogenous protein. (B) Representative IFA images of PfGAP45 and PfGAP50 in mock- (No-GlcN) or GlcN-treated PfGAP40^cKD^ parasites. BF, bright field; blue, nucleus; green, rabbit anti-PfGAP45 or anti-PfGAP50. Scale bars: 5 μm. (C) Western blot analysis of PfGAP40 KD parasites shows the dominant expression of glideosome-associated proteins PfGAP40, PfGAP45, and PfGAP50 in matured schizonts (left) and a dramatic reduction of PfGAP45 and PfGAP50 expression in schizonts upon PfGAP40 knockdown (right). PfGAP40^cKD^ parasites were tightly synchronized and harvested at 0, 6, 12, 24, 36, 40, and 44 hpi, following mock or GlcN treatment and incubated with anti-PfGAP45, anti-PfGAP50, anti-Ty, and anti-aldolase antibodies. Molecular mass in kDa. Representative results from three biological replicates are shown.

In Toxoplasma gondii, TgGAP40 is a polytopic IMC protein, and coimmunoprecipitation (co-IP) analysis formally established the association of this protein with the MyoA-MLC1-GAP45-GAP50 complex (25). Comparable to previous reports, our co-IP assays performed with anti-Ty antibodies precipitated two gliding-associated proteins, namely, PfGAP45 and PfGAP50 (Fig. 5A). In P. falciparum, PfGAP45 and PfGAP50 play critical roles in assembling and anchoring the motor complex MyoA-MTIP to the IMC (21, 25, 56). We therefore examined the localization of these two glideosome proteins in matured schizonts (44 hpi) of the PfGAP40^cKD^ KD parasites. In PfGAP40^cKD^ parasites cultured without GlcN, the fluorescence signals of PfGAP45 and PfGAP50 were distributed at the periphery of individual merozoites, consistent with their proposed location at the IMC (Fig. 5B). However, the fluorescence signals of both PfGAP45 and PfGAP50 were degraded rapidly at 44 hpi following PfGAP40 KD (Fig. 5B). Although small amounts of PfGAP40, PfGAP45, and PfGAP50 could be detected since the early blood stage, consistent with previous results, the strongest expressions of these glideosome complex components were detected from the late schizont stage. Comparable to that observed in IFA analysis, the expressions of PfGAP45 and PfGAP50 were reduced dramatically in schizont stages (40 and 44 hpi) upon PfGAP40 level reductions (Fig. 5C). These results collectively indicate that PfGAP40 is necessary for the efficient expression of PfGAP45 and PfGAP50 in the schizont stage.

### Phosphorylation of PfGAP40 at C-terminal serine sites is not required for IMC localization.

**FIG 6:**
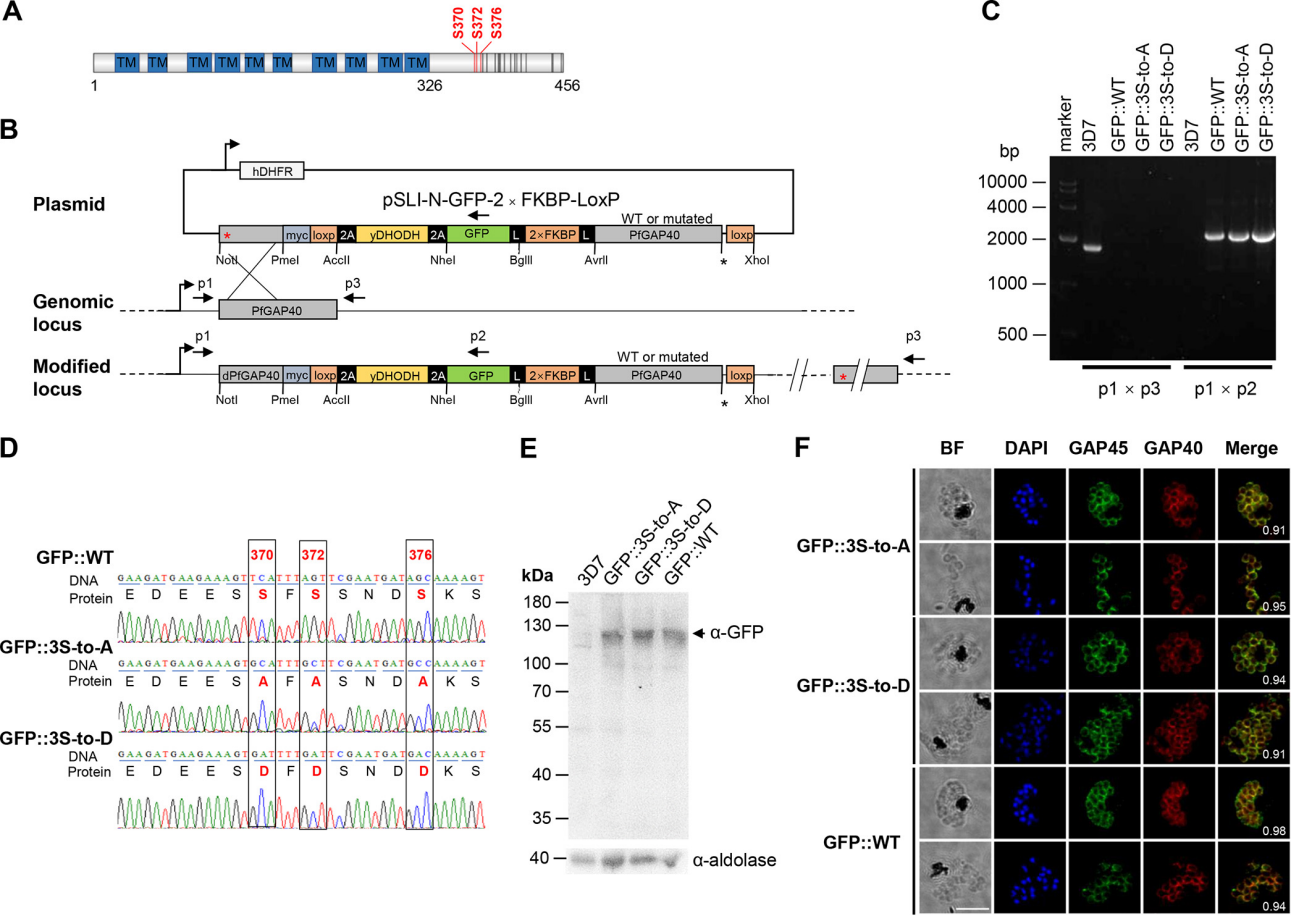
Mutations of phosphorylation sites do not affect the localization of GFP-tagged PfGAP40 proteins to the parasite periphery. (A) Schematic illustrating PfGAP40 with phosphorylation sites that have been identified by various studies (35, 45, 48, 57). The phosphorylation sites are marked with lines, with the sites investigated in this study highlighted with red lines and sites not investigated highlighted in black. TM, transmembrane domain. (B) Illustration depicting the strategy to mutate S370, S372, and S376 sites of PfGAP40 to alanine and aspartic acid using selection-linked integration (SLI) method. The location of PCR primers used for genotyping are indicated; hDHFR, human dihydrofolate reductase; yDHODH, yeast dihydroorotate dehydrogenase; GFP, green fluorescent protein; FKBP, FK506-binding protein. (C) Diagnostic PCR of PfGAP40 phosphorylation mutants. The expected sizes of the PCR products that represent the integration of the native locus (p1×p3: WT, 1,721 bp; mutant, null) and the modified locus (p1×p2: WT, null; mutant, 2,868 bp) are indicated. (D) PCR products encompassing the modified locus were sequenced. An image of the chromatogram reveals successful editing of the locus of interest (marked with black boxes). (E) Western blot analysis of the transgenic mutants. The lysates were probed with anti-GFP antibody, which recognized a band of 132.5 kDa, corresponding to the GFP-2×FKBP-PfGAP40 fusion protein in all three mutants (GFP::WT, GFP::3S-to-D, and GFP::3S-to-A). The blots were also probed with an anti-aldolase antibody to serve as a loading control. (F) IFA analysis of matured schizonts and merozoites from three GFP::PfGAP40 mutants (GFP::WT, GFP::3S-to-D, and GFP::3S-to-A). Specific GFP-2×FKBP-PfGAP40 fusion proteins were probed with anti-PfGAP45 (green) and anti-GFP (red) antibodies. BF, bright field. Nuclei are stained with DAPI (blue). Scale bar: 5 μm.

Global phosphoproteomic studies using merozoites released from synchronized P. falciparum schizonts identified the phosphorylation of a number of glideosome proteins (35, 45, 48, 57), among which PfGAP40 is highly enriched in phosphorylation sites (35). Twenty-two predicted Ser/Thr phosphorylation sites are located in the C terminus from 327 to 456 aa in PfGAP40 (35, 43, 45), suggesting that this region may be important for regulating PfGAP40 function (Fig. 6A). Three sites (S370, S372, and S376) are located within a conserved sequence (S^370^FS^372^SNDS^376^) of *Plasmodium* sp. GAP40 proteins and were identified as the substrates of protein phosphatase 6 (data not shown). In addition, these three sites showed top predicted phosphorylation scores using the NetPhos3.1 software (Fig. 6A). To test the role of phosphorylation on PfGAP40 function, we mutated the residues S370, S372, and S376 in the N-terminal GFP-2×FKBP-tagged PfGAP40 transgenic parasite (GFP::WT) to either an alanine (phospho-null, GFP::3S-to-A) or aspartic acid (phospho-mimetic, GFP::3S-to-D) using the selection-linked integration (SLI) system (58) (Fig. 6B). Parasite lines GFP::WT, GFP::3S-to-A, and GFP::3S-to-D were generated successfully, and the desired modifications were confirmed by PCR and sequencing (Fig. 6C and D). The anti-GFP antibody recognized a band of 132.5 kDa corresponding to GFP-2×FKBP-tagged PfGAP40 in transgenic parasites (Fig. 6E). Meanwhile, immunofluorescence assays and live fluorescent imaging of mature schizonts and released merozoites revealed that all GFP-2×FKBP-tagged PfGAP40 mutants were expressed and targeted correctly to the P. falciparum IMC (Fig. 6F; see Fig. S3 in the supplemental material). In addition, we generated two other PfGAP40 mutants in which S370, S372, S376, S405, S409, S420, and S445 were mutated to alanine (GFP::7S-to-A) or aspartic acid (GFP::7S-to-D) (see Fig. S4A to D in the supplemental material). The GFP::7S-to-A and GFP::7S-to-D mutants showed proper localization of PfGAP40 to the periphery of merozoites in mature schizonts and were colocalized with PfGAP45 (Fig. S4E and F). Taken together, these results indicate that the phosphorylation sites investigated in our study do not seem to impact the targeting of PfGAP40 to the IMC and the assembly of the glideosome.

### Phosphorylation of C-terminal serine sites in PfGAP40 is important for RBC invasion.

**FIG 7:**
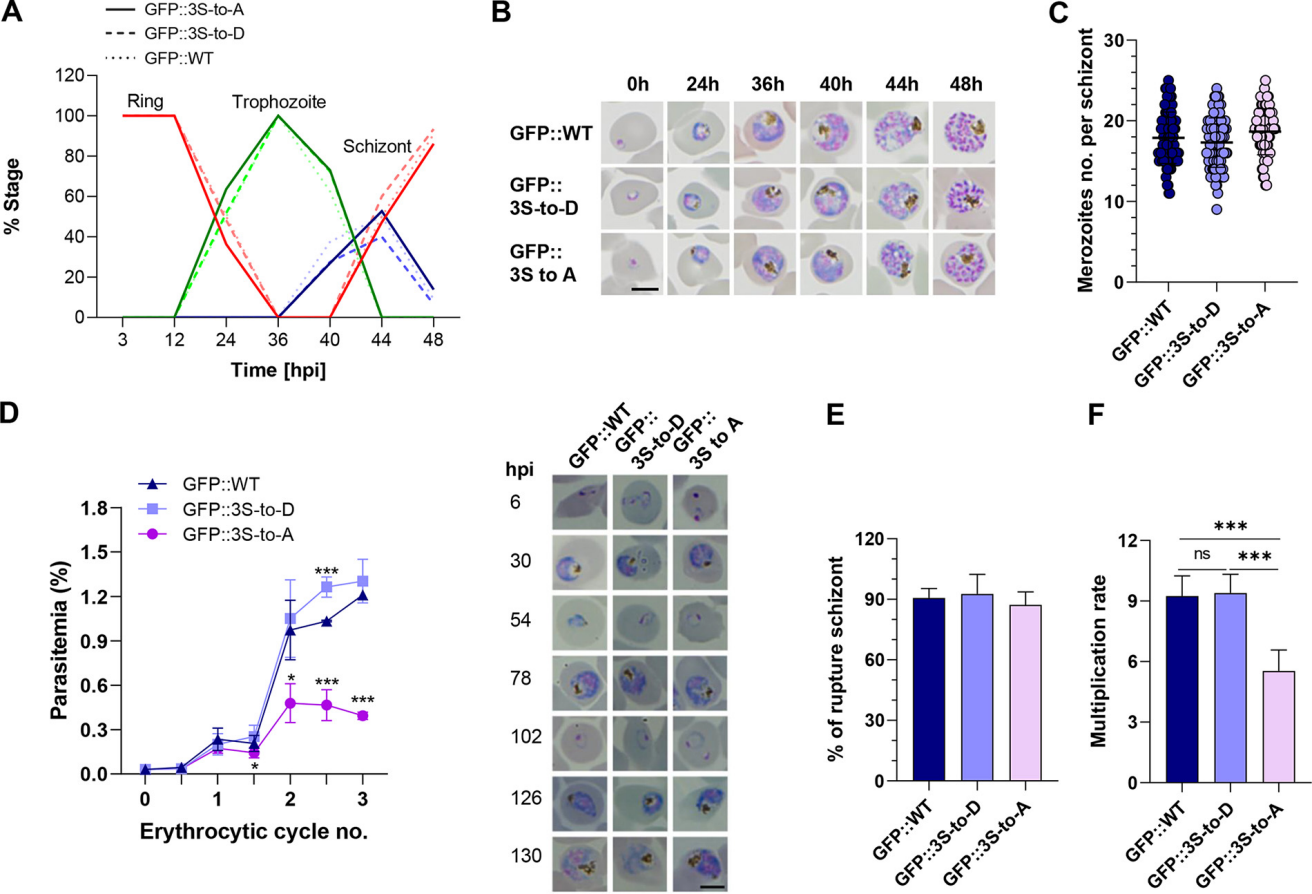
Phosphorylation of the PfGAP40 protein at S370, S372, and S376 sites is critical for its role in host RBC invasion. (A and B) The intraerythrocytic development of GFP::WT, GFP::3S-to-D, and GFP::3S-to-A mutants was compared by monitoring Giemsa-stained thin blood smears using tightly synchronized ring-stage parasites at the indicated times postinvasion. Scale bar: 5 μm. (C) Merozoite number per schizont. (D) Growth curve showing the replication of GFP::3S-to-D and GFP::3S-to-A parasites relative to GFP::WT parasites. Parasitemias were quantified by Giemsa-stained blood thin smears. Error bars indicate ±SD (*n* = 3). Representative images from each detected time point are shown in the right panel. Scale bar: 5 μm. Values are means ± SD. Unpaired *t* test. *, *P < *0.05; ***, *P < *0.001. (E) Percentage of ruptured schizonts. (F) Multiplication rate. Data are the average multiplication rate calculated from three successive cycles (mean ± SD, *n* = 3). Representative results from 3 independent experiments are shown. Unpaired Student's *t* test. ns, not significant. ***, *P < *0.001.

To establish the effects of PfGAP40 phosphorylation on parasite growth, we compared the replication rates of GFP::3S-to-A and GFP::3S-to-D parasites using the GFP::WT mutant as a control. No morphological or growth differences were evident in the first cycle, and all parasites matured to the schizont stage at the same rate with no differences in the number of merozoites produced per schizont (Fig. 7A to C). The parasitemia of GFP::3S-to-D parasites was comparable to GFP::WT parasites by the middle of the next cycle. However, compared with GFP::WT and GFP::3S-to-D parasites, the GFP::3S-to-A parasites had 30.1% and 42.9% reductions in parasitemias, respectively, by the middle of the next cycle (Fig. 7D). When further monitoring cycles 2 and 3, we observed a clear replication defect in the GFP::3S-to-A parasites, demonstrating that the loss of phosphorylation at the C-terminal serine sites of PfGAP40 severely impacted the rate of parasite replication (Fig. 7D). The GFP::3S-to-A mutant did not demonstrate noticeable defects in merozoite egress from the RBC compared with GFP::WT and GFP::3S-to-D mutants (Fig. 7E). However, the multiplication rate was reduced significantly by nearly 41.1% and 42.1% in the GFP::3S-to-A mutant compared with the GFP::WT and GFP::3S-to-D mutants, respectively (Fig. 7F). Moreover, compared with the GFP::WT and GFP::7S-to-D strains, the GFP::7S to A mutant showed more severe reductions (61.7% and 63.5%) in multiplication rates (see Fig. S5 in the supplemental material). Together, our data indicate that the phosphorylation of PfGAP40 at the C-terminal serine sites S370, S372, S376, S405, S409, S420, and S445 is required for efficient RBC invasion.

## DISCUSSION

Malaria parasite invasion of host RBCs is powered by the glideosome and has been the subject of intense research. We characterized the glideosome protein GAP40, which is conserved among Apicomplexa parasites, and demonstrated that it is required for asexual replication and gametocytogenesis in the human malaria parasite P. falciparum. A large-scale screening using the *piggyBac* transposon suggested that PfGAP40 is dispensable for asexual growth in P. falciparum (29). However, the *piggyBac* insertion was located in the 5′ untranslated region (UTR) of the *pfgap40* gene, suggesting that PfGAP40 may still be transcribed and translated. The PfGAP40-Ty signal is visible throughout the IDC, similar to PfGAP50 (23), with an expression pattern unlike other components of the glideosome, such as MyoA, MTIP, GAP45, and most IMC proteins, which disappear following RBC invasion and start transcription in late trophozoites (27). In early stages, namely, ring and trophozoite, PfGAP40-Ty assumes a cytoplasmic location pattern, whereas in mature schizonts and released merozoites, PfGAP40-Ty becomes more abundant and adopts a peripheral location characteristic of the glideosome (23). In both P. falciparum and T. gondii, the IMC appears to originate from the endoplasmic reticulum and an ultrastructural study of PfGAP50 supports that this protein is involved in IMC origins (29, 59).

In apicomplexans, the IMC serves as a scaffold for the assembly of daughter cells during asexual-stage multiplication (60–62). Several reports in T. gondii and P. falciparum showed that conditional deletion of *actin*, *myoa*, *gap45*, and *mlc1/mtip* did not result in defects in IMC formation and integrity, suggesting that these key motor components are not directly involved in IMC biogenesis and stabilization during parasite cytokinesis (25, 63–66). Instead, conditional T. gondii GAP40 knockout (KO) results in a collapsed IMC, and the parasites are unable to form organized daughter cells, implying a critical role of TgGAP40 in stabilization and *de novo* formation of the IMC during intracellular replication (28). Although conditional PfGAP40 KD displayed significantly reduced parasite growth, we observed comparable numbers of merozoites formed in schizonts compared with those control parasites and structural integrity of the IMC in mature schizonts as revealed by both IFA and TEM, thus, indicating that PfGAP40 is not required for IMC biogenesis and stability during cell division. Compared with TgGAP40, PfGAP40 possesses one more TM and a much longer C-terminal region, which may result in topological and functional changes. Furthermore, these phenotypes also suggest that the mechanisms governing cytokinesis may be quite distinct between P. falciparum and T. gondii.

Our observation that PfGAP40 KD did not influence merozoite egress is compatible with recent reports that the actomyosin-based motor is not involved in egress and that *Plasmodium* parasites rely solely on motor-independent mechanisms for egress, such as osmotic pressure and proteolysis (66–68). Time-lapse microscopy and cytochalasin D-based assays revealed that the PfGAP40 KD merozoites could attach to the RBCs but could not invade. Thus, a defect in RBC invasion was the most likely cause of the failure of PfGAP40 KD parasites to propagate.

The glideosome is highly conserved among apicomplexan parasites, and several studies have demonstrated that after the initial attachment, reorientation, and tight junction formation, the merozoite propels itself into the host RBC using the force generated by the actomyosin motor complex of the glideosome (33, 69). The motor complex is assembled to the IMC by the C-terminal region of GAP45 (25, 66, 69) and together with the IMC embedded GAP proteins, GAP40 and GAP50, forms the glideosome. Conditional GAP45 KO results in a deficient MyoA-MTIP assembly to the IMC and a significantly reduced invasion phenotype without affecting GAP50 localization and expression (66). This study confirms that GAP45 is at the core position during glideosome formation and plays a role in stabilizing the glideosome during merozoite invasion. Several studies have demonstrated that GAP50 interacts with PfGAP45 and MTIP and acts as an anchor for the glideosome to the IMC (21, 23, 56). Whether GAP40 is involved in this process is unclear. In T. gondii, although GAP40 was found to interact with MTIP, it is not required for anchoring the MyoA-MTIP-GAP45 complex to the IMC (28). In P. falciparum, the interactome of PfGAP40 contains MyoA, GAP45, GAP50, and MTIP (27). We observed by IFA that the accessibility of PfGAP45 and PfGAP50 to the IMC was significantly reduced in the absence of PfGAP40. These results were further confirmed by Western blotting in which PfGAP40 depletion led to a reduced expression level of PfGAP45 and PfGAP50 in the schizont stage. Collectively, these results indicate that PfGAP40 is essential for stabilizing the expression level of PfGAP45 and PfGAP50 in the schizont stage and is crucial for glideosome function during RBC invasion.

Phosphorylation regulates many cellular processes in P. falciparum, such as schizont egress and RBC invasion (45–47). GAP40 is the most prominent phosphorylated protein in the glideosome in P. falciparum and T. gondii (27, 35, 45–47). Given the total number of phosphorylation sites present in the C-terminal region of PfGAP40, the generation of parasites containing all possible combinations of phosphorylation site mutations is not experimentally feasible. Therefore, we restricted our analysis to the seven serine residues of GAP40 (S370, S372, S376, S405, S409, S420, and S445) that are conserved in *Plasmodium* spp., by substituting serine with either alanine or aspartic acid residues. Several studies highlight the role of phosphorylation in the assembly and function of the glideosome (34, 70). For instance, reducing the phosphorylated forms of PfGAP45 resulted in a significant decrease in its interaction with the actomyosin motor (71). However, in *Toxoplasma*, phosphorylation of MLC1 and GAP45 has no apparent effect on glideosome assembly (34). Here, we found that mutations of the PfGAP40 phosphorylation sites at serines 370, 372, 376, 405, 409, 420, and S445 (GFP:: 7S to A mutant line) did not affect the targeting of PfGAP40 to the periphery of merozoites, thus suggesting that the phosphorylation of these residues of PfGAP40 are not critical for protein trafficking and localization (34). We also demonstrated that PfCRK4-dependent phosphorylation of PfGAP40 at S370, S372, and S376 (43) was not essential for its function in merozoite egress but was required for efficient invasion. Moreover, the GFP::7S-to-A mutant showed a more severe growth phenotype than the GFP::3S-to-A mutant. Previous reports identified PbGAP40 as a substrate for CDPK1 at S448 and S449 sites, and the mutation of these sites does not affect either parasite growth or integrity of the IMC (31, 44). Since the S448 site in PbGAP40 corresponds to S445 of PfGAP40, it is reasonable to propose that the remaining six phosphorylated residues may play a more significant role in asexual proliferation. Although we found that alanine substitutions of the phosphorylated residues significantly impaired merozoite invasion, we did not observe a mislocalized GAP45 signal in these mutant lines. A recent study in *Toxoplasma* proposed that TgGAP45 is responsible for the initial recruitment of the actomyosin motor to the IMC, and subsequent anchoring of the motor in the IMC is dependent largely on IMC-resident proteins, such as TgGAP40 and TgGAP50 (72). Thus, it is possible that the phosphorylation of GAP40 may affect the gliding force generation by the actomyosin motor.

In summary, this study used conditional KD and mutagenesis to dissect the roles played by the P. falciparum glideosome-associated protein GAP40 during RBC invasion and gametocytogenesis. We demonstrate that GAP40 is an essential parasite gene for asexual-stage proliferation in P. falciparum. Although PfGAP40 is not responsible for egress and RBC attachment during merozoite invasion, we determined that this protein is essential for stabilizing the expression levels of PfGAP45 and PfGAP50 in the schizont stage and, therefore, controls the formation of a functional, stable glideosome complex anchored between the PM and the IMC to power merozoite invasion. We found that PfGAP40 also plays a role in gametocyte maturation. Moreover, it was evident from our studies that ablated phosphorylation of PfGAP40 at residues S370, S372, and S376 significantly impaired merozoite invasion. Further work will be required to understand the effect of phosphorylation on various serine sites in PfGAP40 and how the motor force is regulated by this posttranslational modification.

## MATERIALS AND METHODS

### Bioinformatics.

The *pfgap40* genomic sequence (PF3D7_0515700) was retrieved from PlasmoDB (https://www.plasmodb.org). Putative transmembrane domains, phosphorylation sites, and palmitoylation sites were predicted by SMART (http://smart.embl-heidelberg.de/), NetPhos-3.1 (http://www.cbs.dtu.dk/services/NetPhos/), and CSS-Palm (http://bioinformatics.lcd-ustc.org/css_palm/), respectively (73, 74). The schematic figure of the Plasmodium falciparum GAP40 (PfGAP40) protein was drawn by IBS 1.0.3 software (75). The GAP40 proteins were aligned with MUSCLE (76). The secondary structure predictions and sequence similarities of PfGAP40 orthologs in Apicomplexa parasites were performed using ESPript 3.0 software (77). The phylogenetic analyses of PfGAP40 orthologs were performed using MEGA X and Evolview v3 software (78, 79). The 3D structure of PfGAP40 was predicted by AlphaFold (80).

### Plasmid construction.

To generate the pL7-cs-PfGAP40-Ty-*glm*S plasmid, the *pfgap40* fragment (514 to 764 bp) used for homologous recombination (PfGAP40HR) was amplified by overlapping PCR using 3D7 genomic DNA as the template. Primers for PCR amplification are listed in Table S1 in the supplemental material. Briefly, the HR1 region (514 to 1,368 bp) was amplified in two successive steps, as follows: first, the HR1-1 and HR1-2 regions were amplified using primers PfGAP40^cKD^-AsciF1/PfGAP40^cKD^-R1 and PfGAP40^cKD^-AsciF1/PfGAP40^cKD^-R2, respectively; and secondly, the fragments HR1-1 and HR1-2 (primers PfGAP40^cKD^-AsciF1 and PfGAP40^cKD^-R2) were fused to create the final HR1 product. The HR2 region (+1 to +764 bp) was amplified with primers PfGAP40^cKD^-F2 and PfGAP40^cKD^-AflIIR2. The HR1 and HR2 fragments were subsequently assembled by overlapping PCR using primers PfGAP40^cKD^-AsciF1 and PfGAP40^cKD^-AflIIR2 to create the PfGAP40HR fragment, which bears the ApaI and NheI sites and 15-bp overlapping sequences necessary for InFusion clone into the pL6-cs-XU-hDHFR plasmid using the restriction sites AscI/AflII (Table S1). Then, the triple Ty and *glm*S sequences (3×Ty-*glmS*) were amplified using primers PfGAP40-3Ty+*glmS*.F and PfGAP40-3Ty+*glmS*.R and cloned into the ApaI and NheI sites of the bypass plasmid pL6-cs-PfGAP40-HR (Table S1). PCR was done with KOD-plus neo (Toyobo, Osaka, Japan) and cloned using the In-Fusion HD cloning kit (Clontech, CA) and Escherichia coli XL10-Gold ultracompetent cells (Clontech). Guide-RNA cloning also used the In-Fusion HD cloning kit (Clontech) with the 20-bp small guide RNA (5′-TTGCTTTGATGAATAATTTC-3′) surrounded by the 15-bp overlapping sequence from the pL6-cs-PfGAP40-HR plasmid necessary for InFusion cloning to generate the pL7-cs-PfGAP40-Ty-*glm*S plasmid (Table S1). The pUF1-BSD-Cas9 plasmid with the BSD selection cassette modified based on pUF1-Cas9 was used for Cas9 expression (49). The final construct of pL7-cs-PfGAP40-Ty-*glm*S was confirmed by sequencing and restriction enzyme analysis.

The 3D7/PfGAP40-phosphorylation site mutant lines were generated using an SLI-based gene-editing approach (58). To obtain the N-terminal PfGAP40 fusion construct, the pSLI-N-Sandwich-loxP (PfK13) plasmid (a kind gift from Tobias Spielmann) was modified as follows: the PfK13 N-terminal sequence was replaced with 639 bp of the N-terminal region (nucleotide positions 4 to 642 bp) of *pfgap40* amplified using primers PfGAP40-HRF1 and PfGAP40-HRR1 (Table S1). This amplified fragment of *pfgap40* was cloned with NotI/PmeI into pSLI-N-Sandwich-loxP (PfK13), thus resulting in the pSLI-N-Sandwich-loxP-PfGAP40(Nter) bypass plasmid. The recoded PfK13 sequence was replaced with the entire open reading frame (ORF) of *pfgap40*, excluding the STOP codon, to yield the vector pSLI-N-Sandwich-PfGAP40^WT^-loxp. The point mutations (serine 370/372/376 to alanine [3S-to-A] and to aspartic acid [3S-to-D], and S370/372/376/405/409/420/445 to alanine [7S-to-A] or aspartic acid [7S-to-D]) were introduced into the *pfgap40* ORF of the pSLI-N-Sandwich-loxP-PfGAP40 plasmid using a Q5 site-directed mutagenesis kit (New England BioLabs, MA) with the primers listed in Table S1, resulting in the final transfection constructs pSLI-N-Sandwich-loxP-PfGAP40^3S-to-A^, pSLI-N-Sandwich-loxP-PfGAP40^3S-to-D^, pSLI-N-Sandwich-loxP-PfGAP40^7S-to-A^, and pSLI-N-Sandwich-loxP-PfGAP40^7S-to-D^. The final constructs were confirmed by sequencing and restriction enzyme digestion analysis.

### Parasite culture, transfection, and genotyping of transgenic lines.

The P. falciparum strain 3D7 was cultured in RPMI-based medium (ThermoFisher Scientific, MA) containing O^+^ human RBCs at 2.5% hematocrit and 0.5% AlbumaxII lipid-rich bovine serum albumin (ThermoFisher) and was maintained under 5% CO_2_, 3% O_2_, and 92% N_2_ conditions at 37°C as described (81). For parasite tight synchronization, matured schizonts were isolated from uninfected RBCs using 40%/70% Percoll and allowed to rupture and invade erythrocytes for 3 h. Unruptured schizonts were eliminated using 5% sorbitol treatment as described (82). All transgenic P. falciparum parasites used in this work were based on the 3D7 or NF54 clone. To generate the PfGAP40^cKD^ parasite line using the CRISPR-Cas9 system (49), cotransfection was performed using ~50 μg each of pL7-PfGAP40-Ty-*glmS* and pUF1-Cas9 plasmid in uninfected RBCs followed by the addition of matured schizonts and selected with 2.5 nM WR99210 (MedChemExpress [MCE], LA) and 2.5 μg/mL blasticidin-S (ThermoFisher). To obtain the PfGAP40 phosphorylation site mutant lines (GFP::WT, GFP::3S-to-A, GFP::3S-to-D, GFP::7S-to-A, and GFP::7S-to-D), transfection was performed in uninfected RBCs followed by the addition of mature schizonts using 100 μg of pSLI-N-Sandwich-PfGAP40^WT^-loxp, pSLI-N-Sandwich-loxP-PfGAP40^3S-to-A^, pSLI-N-Sandwich-loxP-PfGAP40^3S-to-D^, pSLI-N-Sandwich-loxP-PfGAP40^7S-to-A^, and pSLI-N-Sandwich-loxP-PfGAP40^7S-to-D^ plasmids, respectively. The transgenic parasites were obtained using 2.5 nM WR99210 (MCE) and subsequently treated with 1.5 μM DSM1 (a dihydroorotate dehydrogenase inhibitor) until a stably growing parasite population was obtained. The drug-selected parasites were subjected to limited-dilution cloning as described (83). Targeted mutagenesis of the *pfgap40* gene in GFP::WT, GFP::3S-to-A, GFP::3S-to-D, GFP::7S-to-A, and GFP::7S-to-D parasites was confirmed by diagnostic PCR of the relevant locus followed by Sanger sequencing using primers listed in Table S1. Sanger sequencing data analysis and visualization were performed using SnapGene 1.1.3 software (Insightful Science, CA).

### Western blot analysis.

Western blotting was performed to detect native or Ty- and GFP-tagged PfGAP40 protein expression, as well as the solubility of PfGAP45 proteins in the parental 3D7 strain and PfGAP40^cKD^, GFP::WT, GFP::3S-to-A, GFP::3S-to-D, GFP::7S-to-A, and GFP::7S-to-D transgenic lines. The parasite samples were collected, washed, and released from RBCs using 0.15% saponin (Sigma-Aldrich, MO) in ice-cold phosphate-buffered saline (PBS; pH 7.4). The parasites were then lysed with the mammalian protein extraction reagent (M-PER; ThermoFisher) supplied with 1×halt protease inhibitor cocktail (ThermoFisher) for 30 min on ice. Following centrifugation at 13,000 rpm for 5 min, the supernatant was collected and measured with a Pierce bicinchoninic acid (BCA) protein assay kit (ThermoFisher). Equal amounts of quantified proteins were incubated for 5 min with NuPAGE lithium dodecyl sulfate (LDS) sample buffer (4×) (ThermoFisher) at 100°C; separated on an 8% or 10% SDS-PAGE gel; transferred to a polyvinylidene fluoride (PVDF) membrane (Millipore, Bedford, USA); blocked with StartingBlock (Tris-buffered saline [TBS]) blocking buffer (ThermoFisher); and probed with anti-Ty1 MAb (BB2; ThermoFisher), rabbit anti-GAPDH pAb (Abcam, Cambridge, UK), rabbit anti-*Plasmodium* aldolase pAb (Abcam), anti-Hsp70 MAb (Abcam), or rabbit anti-PfGAP45 serum (which was custom generated by Genescript against amino acids 1 to 204). Membranes were washed, then incubated with the relevant horseradish peroxidase (HRP)-conjugated secondary antibodies (ThermoFisher), and detected using an enhanced chemiluminescence (ECL) kit (Invitrogen, MA) on a Tanon 4200 system (Shanghai, China).

### Fluorescence microscopy assay.

The subcellular localization of the PfGAP40 protein in P. falciparum was observed under a fluorescence microscope as described (84). Briefly, the parasites were fixed with 4% paraformaldehyde and 0.0075% glutaraldehyde in PBS for 30 min at room temperature (RT). Fixed parasites were washed and permeabilized with PBS containing 0.1% Triton X-100 for 10 min on ice, washed 3 times with 1× PBS, rinsed with PBS containing 0.1 mg/mL of sodium borohydride, and blocked in PBS containing 5% skimmed milk (ThermoFisher) prior to staining. Then, the samples were incubated at RT for 2 h with a combination of the Ty1 tag monoclonal antibody (MAb) BB2 (1:500; ThermoFisher), and either a rabbit anti-PfGAP45 serum, rabbit anti-Pfs16 serum (1:500; made in our laboratory), rabbit anti-EXP2 serum (1:500; a gift from Tsuboi, Ehime University, Japan), rabbit anti-α-tubulin II serum (1:500; made in our laboratory), or rabbit anti-PfG377 serum (1:500; made in our laboratory). The samples were then washed 3 times with 1× PBS (pH 7.4), and the parasites were incubated a further 1 h in Alexa Fluor 488- and 594-conjugated secondary antibodies (ThermoFisher). After 5 to 6 final washes in 1× PBS, the samples were mounted with ProLong diamond antifade mountant with 4’,6-diamidino-2-phenylindole (DAPI; ThermoFisher) and imaged using a Leica Stellaris 5 confocal microscope with a 100× oil lens objective. Postprocessing and image analysis were performed using Adobe Photoshop and Image J software. At least 20 images were captured for each colocalization experiment, and Pearson’s correlation coefficients were calculated.

### Live-cell imaging.

Live-cell imaging was performed as described (85). Briefly, after synchronization with 5% sorbitol at the early ring stage (3 hpi), parasites were allowed to mature; schizonts (42 hpi) were purified from uninfected RBCs using 40%/70% Percoll as described above and treated with 10 μM E64 cysteine protease inhibitor (ThermoFisher) for 3 to 4 h to prevent egress; and the parasites were incubated with fresh RBCs at 2% hematocrit in RPMI complete medium containing dyes from the Image-IT live plasma membrane and nuclear labeling kit (Thermofisher) at 37°C for 10 min. The labeled parasites were washed twice in PBS and then plated on a cell imaging cover glass (Eppendorf, Hamburg, Germany) coated with poly-l-lysine (Solarbio, Beijing, China). Live-cell imaging of the parasites was performed on a Leica Stellaris 5 confocal microscope. The sample chamber was maintained at 37°C and supplied with a humidified atmosphere under 5% CO_2_.

### Time-lapse video microscopy.

To visualize the egress to invasion of PfGAP40^cKD^ merozoites in both GlcN-treated and control cultures, time-lapse video microscopy was performed as described with some modification (86). Briefly, tightly synchronized matured schizonts (42 hpi) were purified using a 40%/70% Percoll gradient (30 min, 3,300 rpm), followed by a wash step performed three times with RPMI 1640 medium (ThermoFisher). Then the cell density was adjusted to 1 × 10^5^ cell/mL with RPMI 1640 medium and loaded onto 35-mm Nunc EasYDish dishes (ThermoFisher). The dishes were incubated for 10 min at 37°C to allow the parasite-infected RBCs to attach to the bottom. The RPMI 1640 medium was removed and replaced with complete medium for P. falciparum culture prewarmed to 37°C, and then parasites were observed using a Leica Stellaris 5 confocal microscope. The mock-treated cultures were stained with 1 μg/mL Hoechst 33342 stain (ThermoFisher) for 5 min before imaging.

### Electron microscopy.

The PfGAP40^cKD^ parasites were treated at the ring stage (3 hpi) with either 2.5 mM GlcN or an equal volume of RPMI 1640 and allowed to develop to the matured schizont stage. The matured schizonts (44 hpi) were purified on a Percoll gradient, washed with PBS, and fixed for 24 h at 4°C with 3% glutaraldehyde and 2% paraformaldehyde in 0.1 M phosphate buffer. Schizonts were washed again, pelleted, and embedded in LR white resin (Sigma) at 50°C for 24 h after dehydration in a graded ethanol series (20%, 50%, 70%, 90%, and 100%, twice each). Next, 70-nm sections were prepared using a Leica electron micrograph (EM; UC7). Sections were poststained with 0.2% (wt/vol) uranyl acetate and 4% (wt/vol) lead citrate (Electron Microscopy Sciences [EMS]) and observed at 120 KV on a Hitachi HT7700 transmission electron microscope (Hitachi, Tokyo, Japan).

### Growth phenotype analysis.

All phenotype assays were conducted in three replicates, and the slide reading was performed in a blind manner. The growth assays were performed as described (87). Briefly, the highly synchronized ring-stage parasites were adjusted to ~0.02% parasitemia and 2% hematocrit and divided into 24-well plates in triplicate for each group. Parasitemia was measured every 24 h for 7 days (3 life cycles) using Giemsa-stained thin blood smears by counting parasites in at least 3,000 RBCs. The invasion and egress assays were performed as described earlier with modifications (38). Briefly, tightly synchronized matured schizonts were isolated as described above and resuspended in complete culture medium, and fresh RBCs were added to obtain a parasitemia of 0.5% in a 24-well plate with 2% hematocrit. The parasite cultures were kept at 37°C with gentle shaking. After 12 h of incubation, the numbers of ring-stage and schizont parasites were determined by counting at least 2,000 cells per condition in randomly selected fields on Giemsa-stained blood thin smears. The numbers of ruptured schizonts and new rings formed per ruptured schizont were calculated with the following equations: ruptured schizonts = [(no. of schizonts at 0 h/no. of RBCs at 0 h) – (no. of schizonts at 12 h/no. of RBCs at 12 h)]/(no. of schizonts at 0 h/no. of RBCs at 0 h) and no. of rings per ruptured schizont = [(no. of rings at 12 h/no. of RBCs at 12 h) – (no. of rings at 0 h/no. of RBCs at 0 h)]/[(no. of schizonts at 0 h/no. of RBCs at 0 h) – (no. of schizonts at 12 h/no. of RBCs at 12 h)].

To compare gametocyte development between the GlcN-treated and mock-treated PfGAP40^cKD^ parasites, tightly synchronized schizont-stage cultures were adjusted to a parasitemia of 0.3% with 6% hematocrit and cultivated in 15 mL of complete medium at 37°C. This time point was designated the first day post-gametocyte induction. The medium was exchanged daily and increased to 25 mL when the parasitemia reached 5%. Cultures were maintained in culture medium for 12 days. The asexual parasites were eliminated by *N*-acetyl-d-glucosamine (Sangon Biotech) from day 6 post-gametocyte induction when the induced parasites were at the ring stage and the parasitemia reached about 13%. The cultures of PfGAP40^cKD^ parasites were treated with either GlcN or RPMI 1640 at day 7 postinduction for 7 days until day 12 post-gametocyte induction. Samples were taken for Giemsa blood thin smear preparation every 24 h starting from day 6 post-gametocyte induction. The gametocytemia was counted per 5,000 RBCs, and gametocyte stages I–V were recorded at different time points. Mature male and female gametocytes were differentiated using morphological characteristics of mature gametocytes in Giemsa-stained blood thin films (88). The sex ratio of males to females was determined by counting at least 500 gametocytes on each slide.

### Parasite attachment assay.

Attachment assays were performed as described with slight modifications (89). Briefly, PfGAP40^cKD^ parasites were tightly synchronized and split into 2 × 20-mL (5% parasitemia and 2.5% hematocrit) lots, and one was treated with GlcN. The parasites were allowed to mature to schizonts (~40 h) and were maintained in 50 U/mL of heparin for an additional ~6 h. Subsequently, parasite-infected RBCs were divided into 4 × 10-mL T25 flasks after washing with complete RPMI 1640 along with fresh RBCs and treated with 1 μM cytochalasin D or dimethyl sulfoxide (DMSO; control). All flasks were equilibrated with the appropriate gas mixture and kept on a shaker at 80 rpm at 37°C for 10 h. Then thin blood smears were prepared, fixed with 4% paraformaldehyde and 0.0075% glutaraldehyde in PBS for 30 min at RT, and blocked with 5% skim milk for 1 h at RT, and the parasite nuclei were stained with DAPI. The numbers of attached parasites were counted by microscopically analyzing the DAPI-stained parasites.

### Statistical analysis.

Statistical analyses were performed using Prism 8.0.1 (GraphPad Software, CA). All experiments were analyzed using Student’s *t* test, and a *P* value of <0.05 was considered significant (*, *P < *0.05; **, *P < *0.01; ***, *P < *0.001). The graphs were plotted with mean values from a minimum of 3 biological replicates in most cases.
