# Supplementary material for: Plasmodium falciparum GAP40 Plays an Essential Role in Merozoite Invasion and Gametocytogenesis
Source: Microbiol Spectr. 2023 May 30;11(3):e01434-23. doi: 10.1128/spectrum.01434-23 (PMC10269477; doi:10.1128/spectrum.01434-23)
Supplement: Supplemental file 1 — Legends of Table S1, Fig. S1 to S5, and Movies S1 and S2. Download spectrum.01434-23-s0001.pdf, PDF file, 0.3 MB [file spectrum.01434-23-s0001.pdf]

SUPPLEMENTAL MATERIAL

TABLE S1. Oligonucleotide primers used in this study.

FIG S1. Sequence analysis of GAP40 protein. (A) Sequence alignment of GAP40 from *Plasmodium spp*. Sequences were aligned using the CLUSTALW multiple alignment program. The GAP40 sequence of *P. falciparum* 3D7 (GenBank ID: XP_001351714.1), *P. reichenowi* SY57 (XP_012761670.1), *P. billcollinsi G01* (*P. sp. DRC-Itaito* SPJ09002.1), *P. gaboni* SY75 (XP_018643092.1), *P. ovale* PocGH01 (SCQ16507.1), *P. vivax* SalI (VUZ96488.1), *P. malariae* UG01 (XP_028862064.1), *P. yoelii* 17X (XP_730242.1), *P. chabaudi* AS (XP_746009.2), and *P. berghei* ANKA (XP_034422360.1) were derived from the NCBI website. Red boxes and white characters indicate strict identities; red characters (or black bold character with color scheme "Flashy") indicate similarity in a group; and a blue frame indicates similarity across groups. (B) The phylogenetic tree was generated with MEGA7.0. Bootstrap values (1,000 replicates) were based on neighbor–joining method analysis. (C) Modeling of PfGAP40. The 3D structure of PfGAP40 protein was predicted by AlphaFold implemented in Uniprot.

Fig S2. PfGAP40 is essential for the formation of mature gametocytes in the NF54 strain. (A) Schematic representation of the generation of the transgenic NF54::PfGAP40^{cKD} line. The arrows indicate primers used to verify the integration of a 3×Ty-*glm*S cassette within the *pfgap40* gene locus in the NF54 strain. (B) PCR analysis of genomic DNA from the NF54 parasites (WT) and the NF54::PfGAP40^{cKD} clone (cKD). Predicted DNA fragment sizes with F1 and R2 are 2161 bp from the cKD parasites and 1794 bp from the WT parasites; and with F1 and R1 are 1106 bp from the cKD parasites and null from the WT parasites. (C) Western blot analysis of PfGAP40-Ty protein in cKD parasites using anti-Ty antibody. Detection of *P. falciparum* GAPDH was used as a loading control. (D) Female/male gametocyte ratio on day 12 post-induction. Means ± SD. Unpaired *t* test. ns, not significant. n = 3 technical replicates per experiment, representative of 3 independent experiments

(1000 infected RBCs counted per sample). (E) Daily gametocytemia after day 7 post-gametocyte induction. Means ± SD. Unpaired *t* test. *, $p < 0.05$; **, $p < 0.01$. n = 3 technical replicates per experiment, representative of 3 independent experiments (10,000 infected RBCs counted per sample). (F) Representative images of Giemsa stained blood smears from NF54::PfGAP40^{cKD} parasites cultured with (GlcN) or without 2.5 mM GlcN (No-GlcN) on days 7, 9, 11, and 13. Scale bars, 5 µm.

FIG S3. Live imaging was performed on PfGAP40's phosphorylation sites S370, S372, and S376 mutant lines. Expression of GFP tagged PfGAP40 protein in late schizonts and merozoite of GFP::WT, GFP::3S-to-D, and GFP::3S-to-A as assessed by live cell fluorescence imaging. BF, bright field. The nucleus was labeled with Hoechst 33342. The parasite plasma membrane (PM) was labeled with red-fluorescent Alexa Fluor™ 594 wheat germ agglutinin (WGA). Scale bars, 5 µm.

FIG S4. Functional analysis of PfGAP40 protein with seven phosphorylation mutants at C-terminal region. (A) Schematic representation of PfGAP40 with the relative location of phosphorylation sites within the C-terminal region. The phosphorylation sites investigated here are highlighted in red. (B) Diagnostic PCR analysis confirming successful integration of the GFP and 2×FKBP cassette at the N-terminus of the *pfgap40* gene locus. (C) Western blot analysis of GFP and 2×FKBP tagged PfGAP40 protein in GFP::PfGAP40 transgenic mutants (GFP::WT, GFP::7S-to-D, and GFP::7S-to-A) were performed using anti-GFP antibody. Detection of *P. falciparum* GAPDH was used as a loading control. GFP-2×FKBP-PfGAP40 migrates as an ~132.5 kDa fragment. Note that the additional band at ~160 kDa (*) arises from a cross-reactivity of the anti-GFP antibody, since it is also detected in 3D7 parasites. (D) Sequencing analysis of the seven phosphorylation residues mutated to either alanine or to asparagine in PfGAP40 mutants. Localization of GFP tagged PfGAP40 fusion proteins in the GFP::WT, GFP::7S-to-D, and GFP::7S-to-A mutants was examined by live image analysis (E) and IFA (F), respectively. BF, bright field. For live imaging analysis, the nucleus was labeled with Hoechst 33342. The plasma

membrane (PM) of the parasite was labeled with red-fluorescent Alexa Fluor™ 594 wheat germ agglutinin (WGA). For IFA analysis, GFP tagged PfGAP40 protein (red) was detected using an anti-FKBP antibody. PfGAP45 (green) is a marker for the inner membrane complex. The nucleus was labeled with DAPI. Scale bars, 5 µm.

FIG S5. Phenotype analysis of serine 370, 372, 376, 405, 409, 420, and S445 mutants (GFP::7S-to-A, and GFP::7S-to-D) of PfGAP40 protein. (A) Growth curve showing parasitemia of GFP::7S-to-D, GFP::7S-to-A and GFP::WT parasites, measured over three replication cycles. Parasitemia was quantified by Giemsa stained blood thin smears. Results are means ± SD from three independent experiments, each performed in triplicates. Statistical significance was measured by unpaired *t* test. ***, $p < 0.001$. (B) Percentage of ruptured schizonts. (C) Multiplication rate. Data are average multiplication rate calculated from three successive cycles (mean ± SD, n = 3). *, $p < 0.05$; **, $p < 0.01$; ***, $p < 0.001$.

MOVIE S1. Mock-treated (RRMI 1640) PfGAP40^{cKD} merozoites deform red blood cells, invade, and induce echinocytosis. Time-lapse video microscopy of mock-treated matured PfGAP40^{cKD} parasites undergoing egress, and RBC invasion.

MOVIE S2. GlcN-treated PfGAP40^{cKD} parasites do not deform or invade RBCs but can attach to erythrocyte surface. Cultures of purified schizonts (~50% segmented) were observed by time-lapse video microscopy for 30 min. No RBC invasion is evident. Blue, nucleus stained with Hoechst 33342.
