# Supplementary material for: Plasmodium falciparum GAP40 Plays an Essential Role in Merozoite Invasion and Gametocytogenesis
Source: Microbiol Spectr. 2023 May 30;11(3):e01434-23. doi: 10.1128/spectrum.01434-23 (PMC10269477; doi:10.1128/spectrum.01434-23)
Supplement: Supplemental file 2 — Table S1. Download spectrum.01434-23-s0002.docx, DOCX file, 0.02 MB [file spectrum.01434-23-s0002.docx]

**Table S1 Oligonucleotides used in this study**

| **Oligo name** | **Oligo sequence (5’ to 3’)** |
| --- | --- |
| Primers used for PfGAP40 knockdown construct | |
| PfGAP40^cKD^-SgRNAF1 | TAAGTATATAATATTGTTGCTTTGATGAATAATTTCGTTTTAGAGCTAGAA |
| PfGAP40^cKD^-SgRNAR1 | TTCTAGCTCTAAAACGAAATTATTCATCAAAGCAACAATATTATATACTTA |
| PfGAP40^cKD^-AsciF1 | GCGGCCCTAGTCTAGGGCGCGCCGGAAAGGGTGTTATCGTATGGG |
| PfGAP40^cKD^-R1 | CTGTTTTGATGAGTAGTTCCTGGATAATTTTGATTTTGATTTGAATG |
| PfGAP40^cKD^-R2 | GCTAGCGGGCCCTGCTTGTGAATCAAGCATTTCATGCTGTTTTGATGAGTAGTTCCTGG |
| PfGAP40^cKD^-F2 | GGGCCCGCTAGCTATATTGTGTATTATGCTTATATATAGATGTTTATG |
| PfGAP40^cKD^-AflIIR2 | TTTTTTTACAAAATGCTTAAGATTTCCTTTTTGCATATTATGGG |
| PfGAP40-3Ty+*glmS*.F | CTTGATTCACAAGCAGGGCCCGAAGTACATACTAACCAAGATCC |
| PfGAP40-3Ty+*glmS*.R | ATAATACACAATATAGCTAGCGTCCCCTCCTACATGTTTTTTGG |
| Primers used for genotype PCR of PfGAP40^cKD^ parasite | |
| GAP40-Ty. F1 (F1) | GGTTGTGGAGAAGAATACATG |
| GAP40-Ty. R1 (R1) | CCGTCTTCTTTTCCTATTGAG |
| GAP40-Ty. R2 (R2) | GATTTCCTTTTTGCATATTATGGG |
| Primers used for N-terminal GFP tagged PfGAP40 construct | |
| PfGAP40-HRF1 | TATAGAATACTCGCGGCCGCTAAATGAAGTTTGAAAAGTTTAATATACC |
| PfGAP40-HRR1 | TAACTTCTGCTCGTTTAAACCCAGATGTTGATTGTTATGTAACG |
| PfGAP40-mut-F1 | CTGGAGGTGCAGGTAGACCTAGGATGAAGTTTGAAAAGTTTAATATAC |
| PfGAP40-StoA-R1 | GGCATCATTCGAAGCAAATGCACTTTCTTCATCTTCTTCATTATC |
| PfGAP40-StoA-F2 | GCATTTGCTTCGAATGATGCCAAAAGTTATCAAGGATCACAAGATTATG |
| PfGAP40-StoA-R2 | GTGAATCAAGCATTTCATGTTGCTTTGATGCATAATTTCTGGATAATTTTG |
| PfGAP40-mR3 | GTATGCTATACGAAGTTATAGGCCTTTATGCTTGTGAATCAAGCATTTC |
| PfGAP40-StoD-R1 | GTCATCATTCGAATCAAAATCACTTTCTTCATCTTCTTCATTATC |
| PfGAP40-StoD-F2 | GATTTTGATTCGAATGATGACAAAAGTTATCAAGGATCACAAGATTATG |
| PfGAP40-StoD-R2 | GTGAATCAAGCATTTCATGTTGCTTTGAGTCATAATTTCTGGATAATTTTG |
| PfGAP40-mut-R3 | CTATACGAAGTTATAGGCCTTTATGCTTGTGAATCAAGCATTTC |
| PfGAP40-mutR | CTTTCTTCATCTTCTTCATTATCAGCTGATGACAGG |
| PfGAP40-7StoA-F | AAGATGAAGAAAGTGCATTTGCTTCGAATGATGCC |
| PfGAP40-7StoD-F | AAGATGAAGAAAGTGATTTTGATTCGAATGATGAC |
| Primers used for genotype PCR of GFP::WT, GFP:: 3S-to-A, GFP:: 3S-to-D, GFP:: 7S-to-A and GFP::7S-to-D parasite | |
| p1 | TCTTATAGACATTAACGACTAC |
| p2 | CGTATGTTGCATCACCTTCACCCTC |
| p3 | GTGAATGTATTCTTGTGTGC |
