# Supplementary figures and images for: Plasmodium falciparum GAP40 Plays an Essential Role in Merozoite Invasion and Gametocytogenesis
Source: Microbiol Spectr. 2023 May 30;11(3):e01434-23. doi: 10.1128/spectrum.01434-23 (PMC10269477; doi:10.1128/spectrum.01434-23)

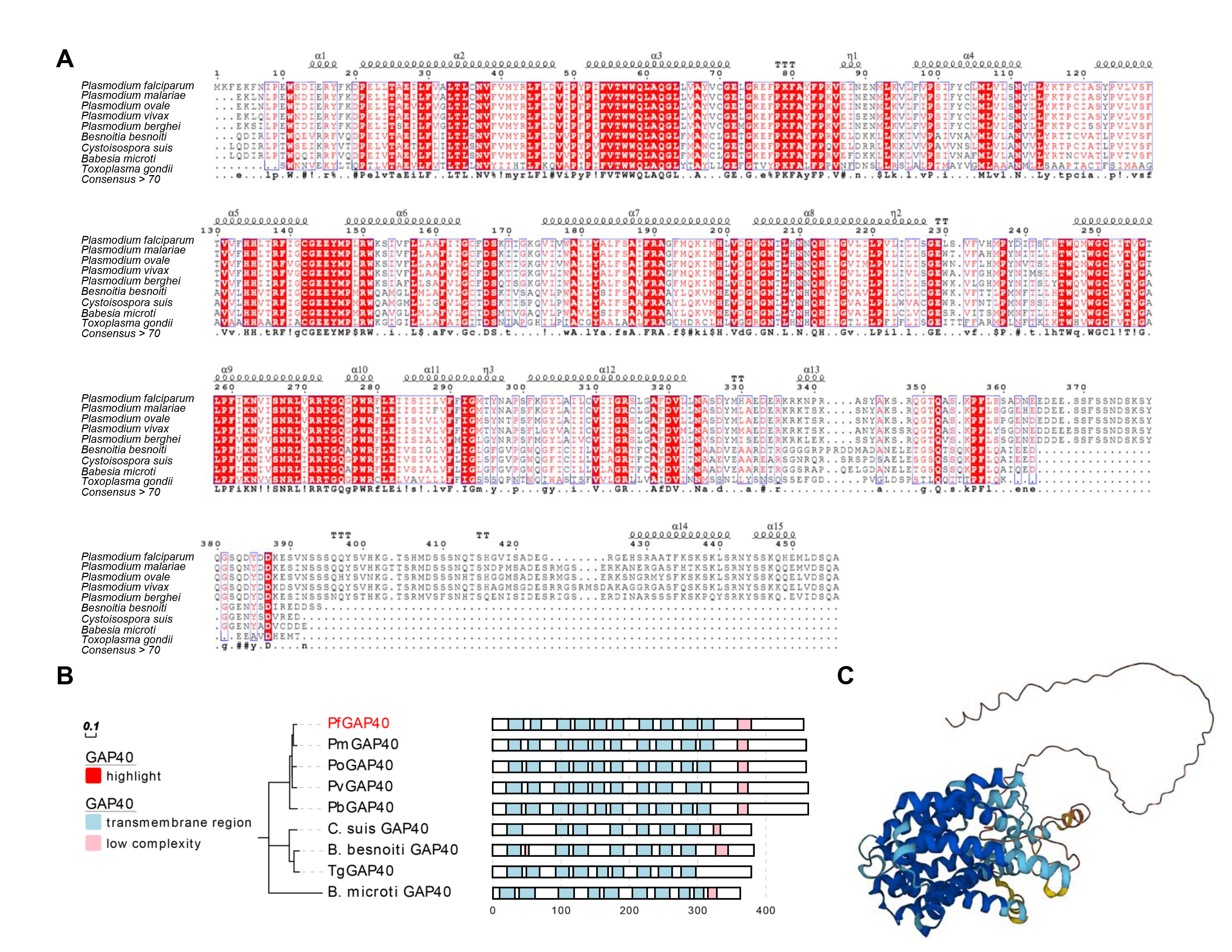

Supplement: Supplemental file 3 — Fig. S1. Download spectrum.01434-23-s0003.tif, TIF file, 6.8 MB [file spectrum.01434-23-s0003.tif]

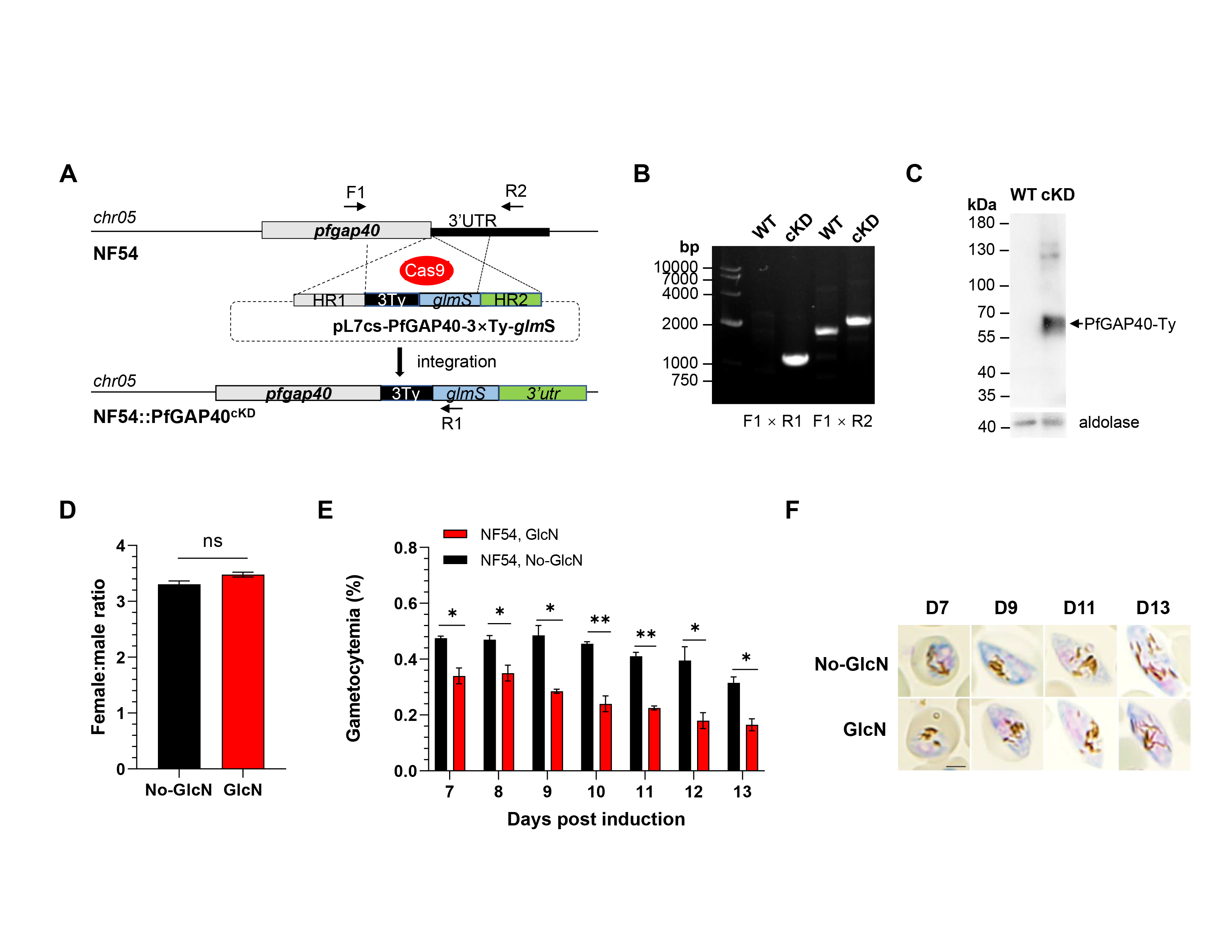

Supplement: Supplemental file 4 — Fig. S2. Download spectrum.01434-23-s0004.tif, TIF file, 0.8 MB [file spectrum.01434-23-s0004.tif]

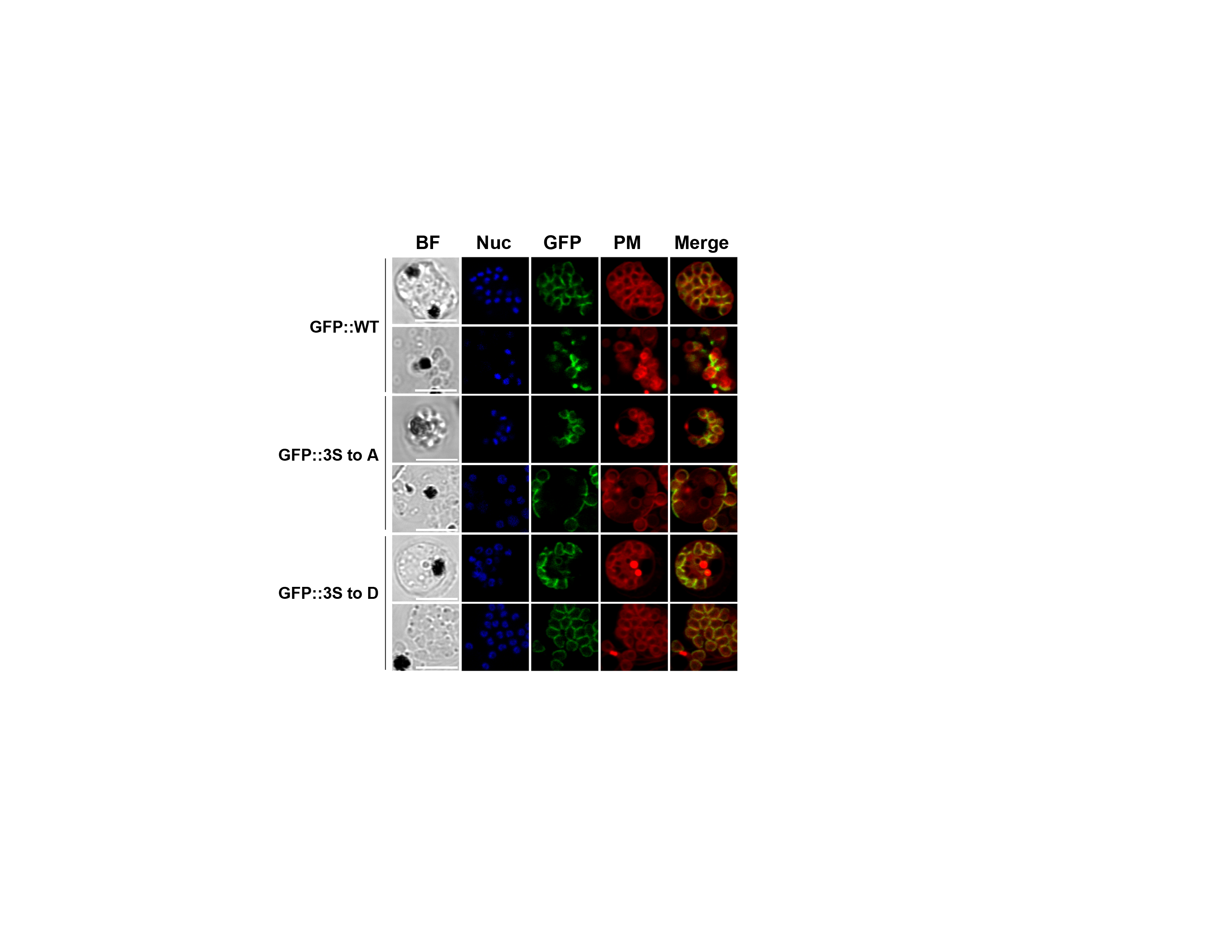

Supplement: Supplemental file 5 — Fig. S3. Download spectrum.01434-23-s0005.tif, TIF file, 1.0 MB [file spectrum.01434-23-s0005.tif]

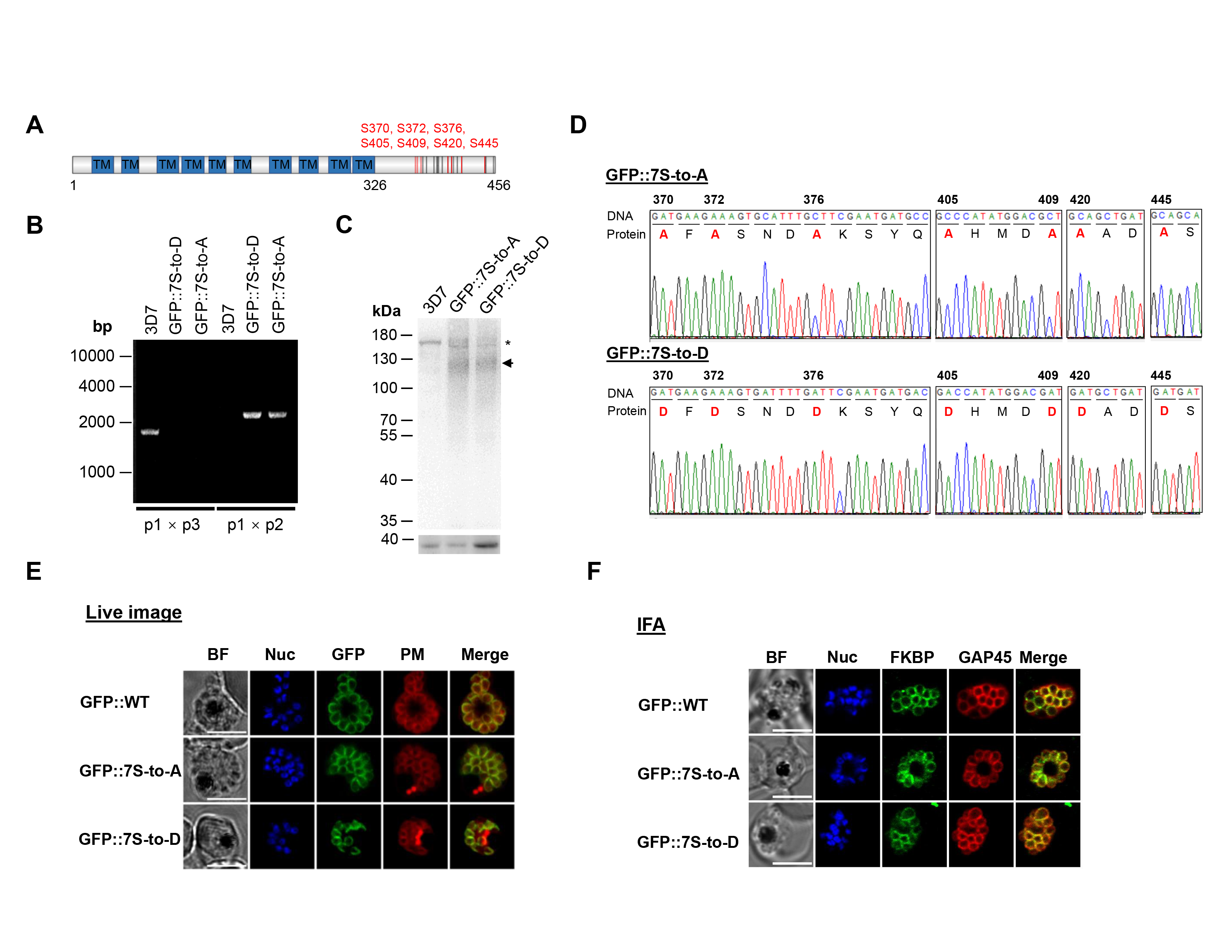

Supplement: Supplemental file 6 — Fig. S4. Download spectrum.01434-23-s0006.tif, TIF file, 1.9 MB [file spectrum.01434-23-s0006.tif]

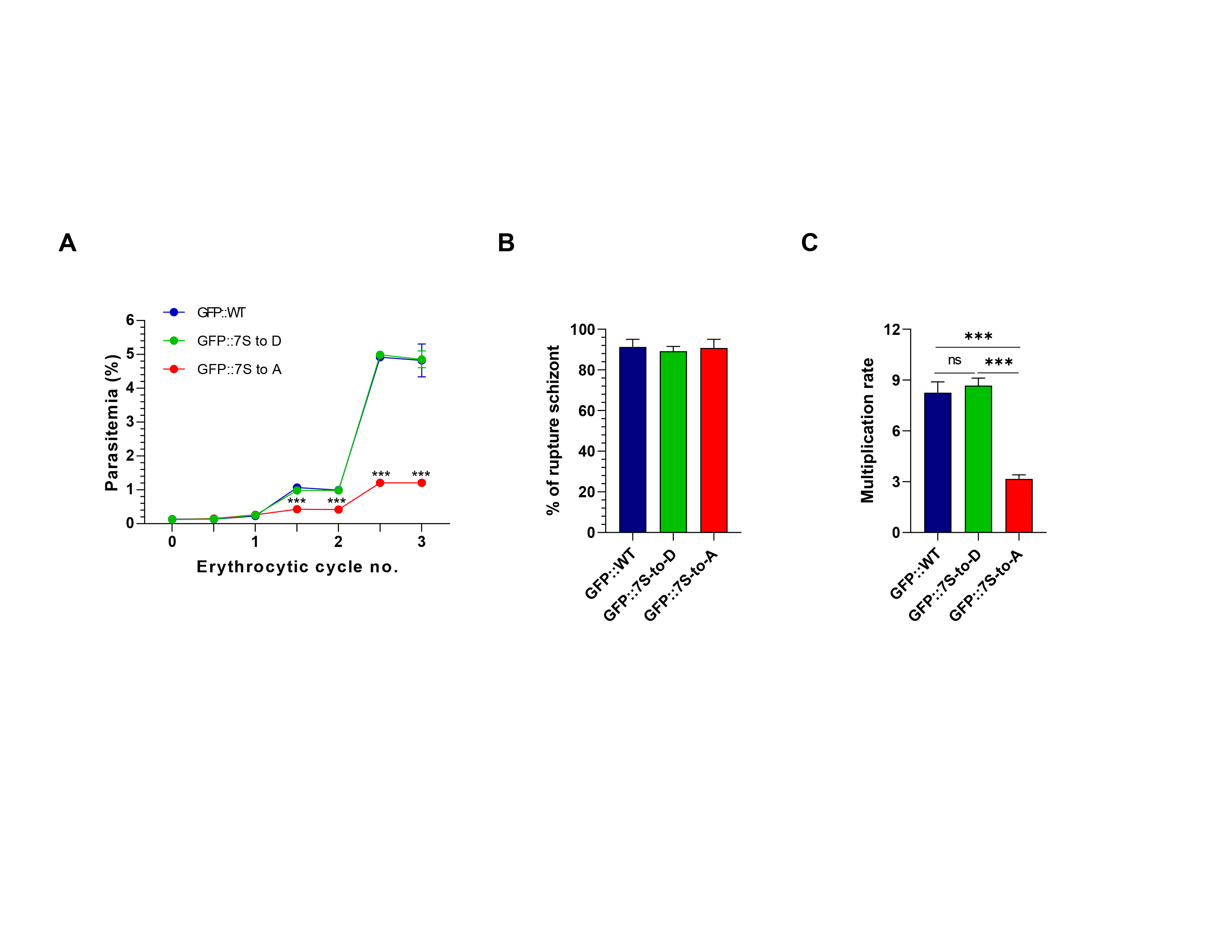

Supplement: Supplemental file 7 — Fig. S5. Download spectrum.01434-23-s0007.tif, TIF file, 0.2 MB [file spectrum.01434-23-s0007.tif]
